# Cellular senescence affects energy metabolism, immune infiltration and immunotherapeutic response in hepatocellular carcinoma

**DOI:** 10.1038/s41598-023-28436-z

**Published:** 2023-01-20

**Authors:** Biao Gao, Yafei Wang, Shichun Lu

**Affiliations:** 1 Nankai University School of Medicine, Nankai University, Tianjin, China; 2 Faculty of Hepato-Pancreato-Biliary Surgery, Chinese PLA General Hospital, Beijing, China; 3 Institute of Hepatobiliary Surgery of Chinese PLA, Beijing, China; 4 Key Laboratory of Digital Hepatobiliary Surgery PLA, Beijing, 10058 China

**Keywords:** Cancer genomics, Cancer metabolism, Cancer, Immunology

## Abstract

Aging is an inevitable consequence of life, characterized by a progressive decline in tissue and organ function and an increased risk of death. There is growing evidence that aging is closely related to tumor development and immune regulation. However, in hepatocellular carcinoma, the relationship between cellular senescence and immune infiltration, energy metabolism, chemokines, and immunotherapeutic response is unclear and needs further study. We first analyzed 274 cellular senescence-associated genes by the NMF algorithm and identified two cellular senescence-associated clusters. Subsequently, we compared the differences between the two clusters, in terms of immune infiltration, energy metabolism, chemokines, and immunotherapeutic response to treatment. We further constructed risk models using cellular senescence-associated signature genes that could effectively identify the two subpopulations. Finally, we validated the validity and robustness of the risk model using an external dataset. We found significant differences in survival prognosis between two cellular senescence-associated clusters. In addition, we found significant differences in immune cell infiltration, expression of energy metabolism-related genes, expression of chemokine-related genes, expression of immune checkpoint-related genes, Tumor Immune Dysfunction and Exclusion between the two clusters. Also, a scoring system associated with cellular senescence was developed and validated as an independent prognostic indicator. It was validated as an independent prognostic factor and immunotherapeutic predictor for HCC. It was validated as an independent prognostic factor and immunotherapeutic predictor for HCC. The cellular senescence-related scoring system was validated as an independent prognostic factor and immunotherapy predictor for HCC, and patients with low CSS were characterized by prolonged survival time. Our study confirmed the relationship between cellular senescence and immune cell infiltration, energy metabolism, chemokines, expression of immune checkpoint-related genes, and response to immunotherapy. This enhances our understanding of cellular senescence and tumor immune microenvironment, energy metabolism, chemokines, and provides new insights to improve immunotherapy outcomes in HCC patients. It provides new insights to improve the outcome of immunotherapy in HCC patients.

## Introduction

Hepatocellular carcinoma (HCC) is a global health problem with an increasing incidence and mortality^1,2^. Despite the increasing use of surgical and local treatments worldwide^3^, it is estimated that about 50–60% of HCC patients will eventually undergo systemic therapy^4^. Since 2017, immunotherapy has been another major breakthrough in advanced HCC with encouraging results. In recent years, with the development of single-cell and second-generation sequencing, multi-omics research, bioinformatics, and tumor microenvironment (TME) research, biomarkers including gene alterations and pathway activations are of great significance for designing proper treatment regimens, and biomarker-driven therapies have shown gratifying benefits^5^. Targeted therapy, immune checkpoint blockers (ICB) therapy, and their combination therapy have shown superior efficacy for HCC in clinical trials. Although understanding of HCC genomics and breakthroughs in targeted therapies and immunotherapy have greatly expanded the therapeutic paradigm, challenges associated with HCC remain elusive. For example, most HCC patients have a low response rate to immunotherapy of 15–25%^6^, which falls far short of clinical needs. Currently, even though the objective response rate of immune checkpoint blockers has almost doubled in combination with targeted drug therapy, more than half of the patients still do not respond^7^. In addition, ICB can cause serious immune-related adverse events (irAEs) and hyperprogression disease (HPD) during anti-PD-1 or anti-PD-L1 therapies in about 8% of HCC cases^8,9^. Biomarker-based patient selection can help maximize efficacy and reduce the number of patients who may not benefit or even be harmed by ICB. Therefore, better immunotherapy prediction tools and biomarkers that accurately predict tumor characteristics are urgently needed to stratify patients and personalize treatment for HCC.

Cellular senescence is a complex stress response that affects cellular function and organismal health. Multiple developmental and environmental factors, such as radiation, oxidative stress, oncogenes, and protein accumulation, can activate genes and pathways leading to senescence^10^. Regardless of the stimuli, senescent cells share some common behaviors, among which the main ones are growth arrest, apoptosis resistance, sustained DNA damage signaling, and heterochromatin modifications^11^. A growing body of research suggests that aging accompanies cellular senescence and that manipulating the biological process of cellular senescence can slow or retard many diseases associated with aging, such as cancer^12,13^. Recent studies have confirmed that aging and cellular senescence affect the immune microenvironment (TME) by promoting the accumulation of multiple types of immunosuppressive cells and activating various risk-related signaling molecules and cytokines, which have a broad impact on the TME and tumor growth^14,15^. At the same time, cellular senescence changes the adaptability of immune cells in the TME, thus altering the efficacy of tumor immunotherapy to some extent^16,17^. Therefore, an improved understanding of the impact of senescence on tumor immunity associated with the invasion and development of HCC is required to frame novel treatment paradigms for tumors.

To provide a comprehensive and systematic understanding of the relationship between cellular senescence and immune infiltration, energy metabolism, chemokines, and immunotherapy response in HCC, we established a cellular senescence scoring system and explored their potential importance as predictive biomarkers for prognosis and immunotherapy response. We further explored the mechanisms by which tumor cell senescence-related genes affect the TME. Subsequently, we validated the robustness and validity of CSS in multiple independent external data sets. This study provided new insights into the regulatory mechanisms of cellular senescence associated with the TME and strategies for HCC immunotherapy.

## Materials and methods

### Data and clinical samples

We collected gene expression data and complete clinical information data from 424 HCC patients using The Cancer Genome Atlas (TCGA, https://portal.gdc.cancer.gov/), which contains 365 tumor tissues and 59 normal tissue samples, as a training set. To validate the robustness of the signature, we downloaded the sample information of tumor tissues from 231 HCC patients from ICGC (https://dcc.icgc.org/) as the validation set. Similarly, bulk RNA sequencing information and clinical information from 220 HCC patients in the GSE14520 dataset were downloaded from GEO as a validation set. Two external independent immunotherapy data were obtained from the IMvigor210 and GSE91061 datasets, respectively. The clinical features of the 365 HCC patients in the training set are summarized in Table 1. The 279 cellular senescence- related genes in this study were obtained from the CellAge database (https://genomics.senescence.info/cells/), which contains manually managed human gene data associated with cellular senescence.

**Table 1:** Clinical characteristics of 365 HCC patients in TCGA.

Characteristics	Samples (n = 365)	Percentage
Gender		
Female	120	32.9
Male	245	67.1
Age		
≥ 60	200	54.8
< 60	165	45.2
Stage		
Stage I	180	49.3
Stage II	91	25.0
Stage III	81	22.2
Stage IV	13	3.6
M		
M0	263	72.1
MX	102	28.0
N		
N0	248	67.9
N1	5	1.4
NX	112	30.7
Grade		
G1	58	15.9
G2	176	48.2
G3	116	31.8
G4	13	3.6

### Identification of cellular senescence clusters by NMF

We used the “non-negative matrix factorization” (NMF) clustering algorithm of the NMF package of R software algorithm to cluster 365 HCC samples based on the expression levels of 279 cellular senescence-related genes to identify distinct cellular senescence clusters. The “brunet” option was selected and 100 iterations were performed for the NMF. We determined the optimal number of clusters based on the apparent coefficients, dispersion coefficients, and silhouette coefficients to determine the optimal number of clusters for the 365 HCC samples.

### Differences between clinical features

We further investigated the relationship between the clusters obtained by NMF clustering and clinical characteristics to determine the effects of cellular senescence-related genes on clinical characteristics.

### Differentially expressed genes (DEGs) and functional enrichment analysis

We used the “limma” package of the R software to perform differential analysis of the different clusters with the screening criteria of *p*-value < 0.05 and logFC > 2 or logFC < − 2. To further investigate the differences in molecular mechanisms between different clusters, we used the "clusterProfiler" package of R software to perform functional enrichment analysis of up-regulated and down-regulated genes separately, with *p*-value < 0.05 considered statistically significant.

### Differences between chemokines, energy metabolism between different clusters

The Molecular Signature Database (MSigDB, http://www.broad.mit.edu/gsea/msigdb/) contains two energy metabolism-related genes set (energy-requiring parts of metabolism and reactome energy metabolism) was used to identify the energy metabolism-related genes. We further downloaded the chemokine-related gene set from TISIDB (http://cis.hku.hk/TISIDB/). To further investigate the relationship between cellular senescence and chemokines and energy metabolism, we compared the expression of chemokines and energy metabolism-related genes among different clusters.

### Evaluation of immune infiltration between different clusters

CIBERSORT is a deconvolution technique that utilizes RNA-Seq data to determine the makeup of immune cells. We used CIBERSORT to analyze RNA expression data from 365 HCC samples, thus comparing the differences between different clusters in terms of immune infiltrating cells. Meanwhile, single sample gene set enrichment analysis (ssGSEA) was implemented to estimate immune cell abundance of each sample in TCGA cohort based on a gene panel marking 28 immune cell types. We further analyzed the RNA expression information of 365 HCC cases using the ESTIMATE function of R software to evaluate the differences between different clusters in StromalScore, ImmuneScore, ESTIMATEScore, and TumorPurity.

### Prediction of immunotherapeutic response

We downloaded somatic mutation data from TCGA for all HCC samples and used the maftools package of R software to analyze different clusters and thus compare differences between clusters in terms of gene mutation and tumor mutation load (TMB). (TMB = (total mutations/total number of tests) * 10^6^). More and more studies have confirmed that MSI can not only predict the chemotherapy efficacy of early colon cancer, gastric cancer, and small bowel cancer but also be a predictor of immunotherapy efficacy of advanced solid tumors^18–21^. Richness and the Shannon Diversity Index were used to describe the diversity of the TCR repertoire. Richness measures the number of unique TCRs in the sample, while the Shannon diversity index reflects the relative abundance of different TCRs. BCR is a B lymphocyte receptor and BCR abundance is a combination of the various BCR isoforms produced in an individual. There is growing evidence that the diversity of peripheral TCR and BCR profiles can serve as a predictive and prognostic biomarker of the immune response in cancer patients. The cancer testicular antigens (CTA) score is used to evaluate tumor immunogenicity, which indirectly reflects the strength of the immunotherapy response. The richness values and Shannon diversity index valves of TCR in the TCGA LIHC patients were obtained from the Pan-Cancer Atlas study^22^. To determine the sensitivity of the two clusters to immunotherapy, we compared the differences in TMB, MSI, TCR richness, BCR richness, and CTA score between the clusters. We further compared the differences in the expression of immune checkpoint-related genes, such as PD-L1, PD1, PD-L2, TIM-3, and TIGIT, among different clusters. The Tumor Immune Dysfunction and Exclusion (TIDE, http://tide.dfci.harvard.edu/), considered to be the best predictor of response to immunotherapy, was used to predict response to immunotherapy among distinct cellular senescence clusters.

### Construction and validation of a cellular senescence scoring system

We further constructed the cellular senescence scoring system to better represent cellular senescence clusters. We used the "limma" package of R (version 4.1.2) to perform differential analysis among clusters with the screening criteria of *p*-value < 0.05 and logFC > 1 or logFC < − 1, to obtain differential expressed genes. Univariate Cox regression analysis was performed to identify cellular senescence-related prognostic genes (*p* < 0.05). Next, the DEGs and prognostic genes were investigated using the R package “veen” to acquire prognostic cellular senescence-related DEGs, and correlations were visualized by the R package “circlize”. To eliminate overfitting, a least absolute shrinkage and selection operator (LASSO) Cox regression analysis was used in conjunction with the "glmnet" package. Finally, we utilized Cox multivariable analysis on the screened variables to find independent prognostic risk factors and construct a cellular senescence-related scoring system. Risk score = $\sum {\left( {{\text{Expi}}\,*\,{\text{Coefi}}} \right)}$. Coefi and Expi denote the risk coefficient and gene expression, respectively. Following that, all patients were classified as high-risk or low-risk based on their median risk score. We validated the robustness of the scoring system using the survival data of 451 HCC patients from the ICGC database and GSE14520.

### Validation was performed in independent external immunotherapy data

To further examine the relationship between cellular senescence and immunotherapy response, we used two external independent immunotherapy data for validation. We calculated risk scores for each patient in both datasets using the same CSS as in the training set and divided all immunotherapy patients into high-risk and low-risk groups based on the median risk score. The proportion of patients who achieved CR/PR (CR: complete response; PR: partial response) or SD/PD (SD: stable disease;PD: progressive disease) following immunotherapy was compared between the two groups to identify the differential in response to immunotherapy between risk groups.

### qRT-PCR confirms overexpression of 3 cellular senescence-related genes in tumor tissues

To verify the mRNA expression levels of two chemokine-related genes and four cellular senescence-related genes in HCC tissue samples, we collected tumor tissue samples and paraneoplastic tissue samples from 12 patients with HCC confirmed by postoperative pathology. We further compared the differences in mRNA expression of two chemokine-related genes and four cellular senescence-related genes in HCC tissue samples and paraneoplastic tissues using qRT-PCR.

### Statistical analysis

Wilcoxon t-test was used to compare differences between categorical variables. Univariate and multivariate Cox regression analyses were used to screen for independent risk factors affecting the prognosis of HCC patients. lASSO regression analysis was used to screen for signature genes for prognosis. A significance criterion of *P* < 0.05 was selected. Benjamini–Hochberg was implemented using the R function p.adjust to alter the *P*-value for multiple testing. R software was used to do all statistical analyses in this study (version 4.1.2).

## Results

### Identification of different cellular senescence-related clusters

**Figure 1:**
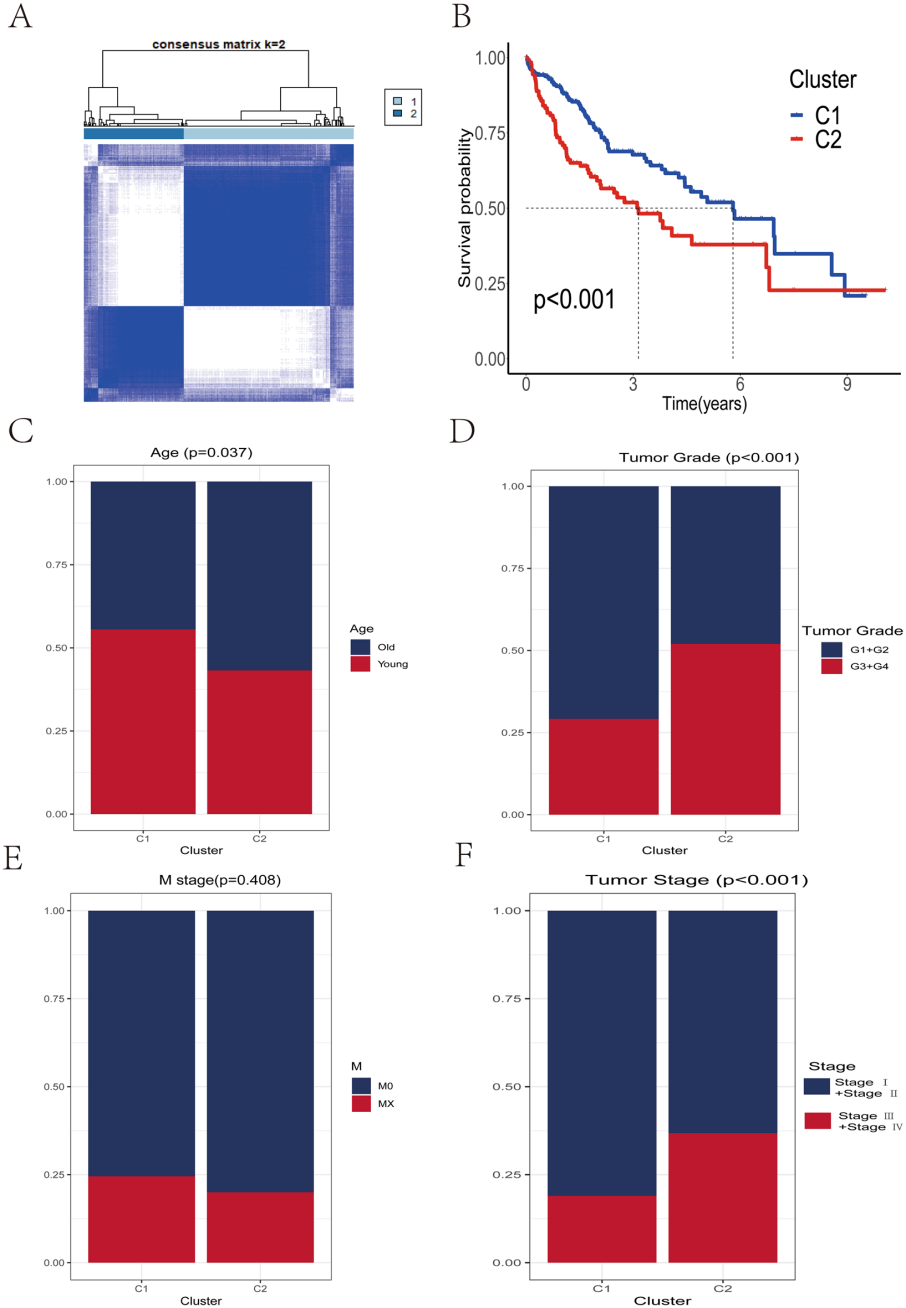
Clustering of HCC patients in the training set and the relationship between the results of each cluster and clinical characteristics and survival. (**A**) The 365 HCC patients in the training set were clustered into Cluster1 (C1) and Cluster2 (C2) based on the expression levels of 279 cellular senescence genes. (**B**) Kaplan–Meier survival analysis showed a significant difference in overall survival time between C1 and C2. (**C**–**F**) The relationship between different clusters and clinical features, including age (**C**), tumor grade (**D**), M stage (**E**) and tumor stage (**F**).

To comprehensively explore the expression patterns of cellular senescence-related genes in HCC, we downloaded the information of RNA sequencing samples and clinical information of 365 HCC patients from the TCGA database as the training set. We downloaded RNA sequencing sample information and clinical information of 461 HCC patients from ICGC database and GEO database as validation set. Based on the expression profiles of 281 cellular senescence-related genes, we stratified 365 HCC patient samples in the training set into two different clusters (233 cases in Cluster 1 (C1), 132 cases in Cluster 2 (C2) by a nonnegative matrix factorization (NMF) algorithm (Fig. 1A). In the training set, the survival of C1 patients was significantly better than that of C2 patients, and the survival curves are shown in Fig. 1B. Subsequently, we further compared the differences between clusters in terms of basic clinical characteristics, and we found that C2 patients had a greater proportion of HCC patients with Stage III and Stage IV compared with C1 patients (Log-rank test, *P* < 0.05), while patients with Tumor Grade G3 and G4 were significantly more represented in C2 than C1 (Log-rank test, *P* < 0.05). Interestingly, we found a higher proportion of old patients (age > 60) in the C2 group (Log-rank test, *P* < 0.05) (Fig. 1C–F). The above results suggest that there is a relationship between the expression of cellular senescence-related gene and clinical characteristics, such as Stage, Age and Tumor Grade.

### Differential gene analysis and functional enrichment analysis

**Figure 2:**
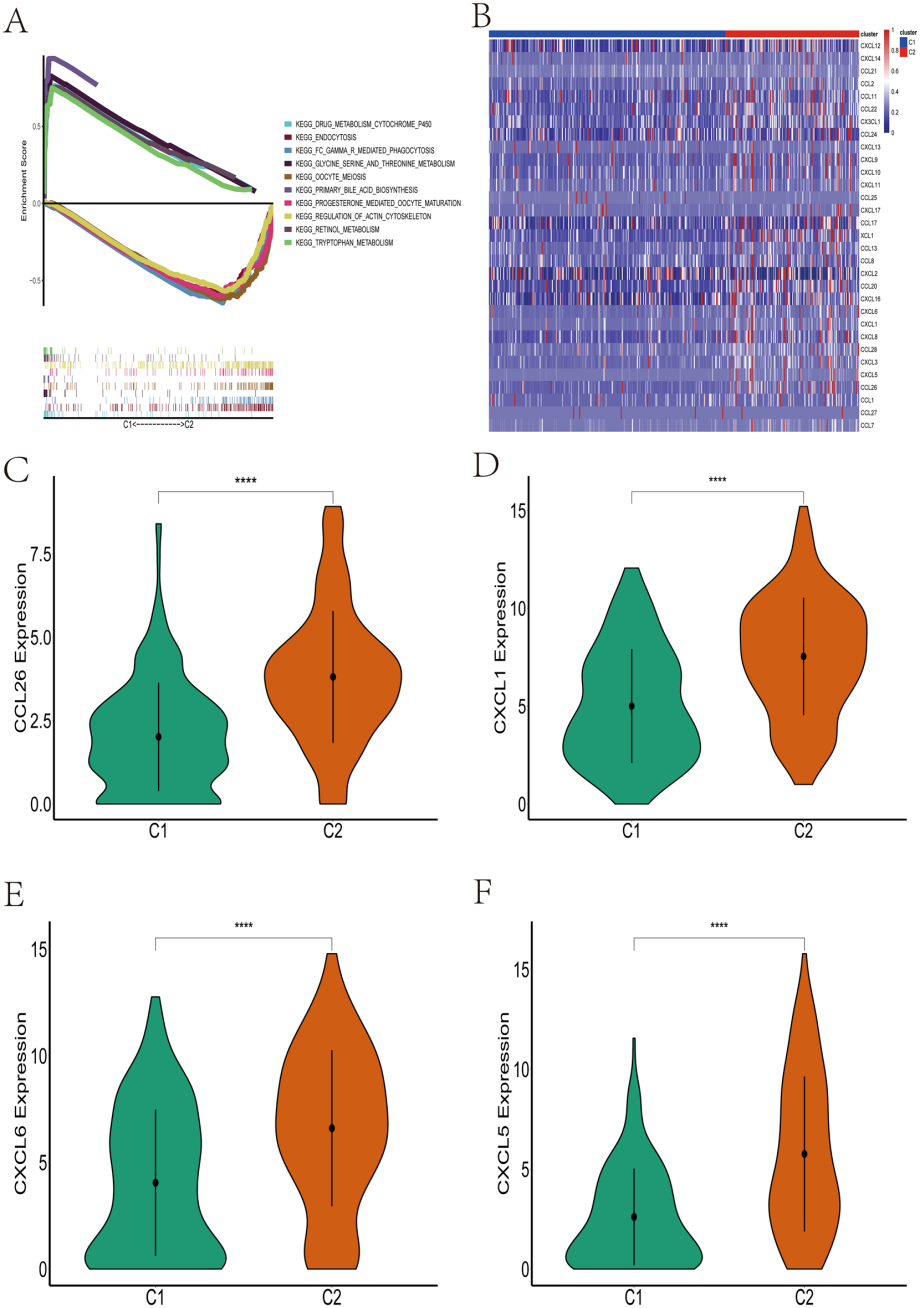
Differential expression of chemokine-related genes among different clusters. (**A**) GSEA enrichment analysis shows that different clusters are involved in different signaling pathways. (**B**) The heatmap demonstrates significant differences in the expression levels of chemokine-related genes between the different clusters. (**C**–**F**) The expression levels of chemokine-related genes were significantly higher in patients in the C2 group than in C1, including CCL26 (**C**), CXCL1 (**D**), CXCL6 (**E**) and CXCL5 (**F**).

To further investigate the differences in gene expression and biological processes involved between different clusters, we further performed differential gene analysis between different clusters, in which 123 genes were up-regulated in C1 and 2253 genes were up-regulated in C2 (Fig. S1A–B). The GO enrichment analysis showed that the biological process (BP) of up-regulated genes in C1 was mainly enriched in small molecule catabolic process and organic acid catabolic process; the most enriched cellular component (CC) was the mitochondrial matrix and apical part of cell (Fig. S1C). The GO enrichment analysis revealed that the biological processes (BP) of upregulated genes in C2 were mainly enriched in positive regulation of cell activation and positive regulation of leukocyte activation; the most enriched cellular components (CC) were external side of plasma membrane and plasma membrane signaling receptor complex (Fig. S1D). KEGG functional enrichment analysis showed that in C1 upregulated genes were mainly enriched in Metabolism of xenobiotics by cytochrome P450 and Drug metabolism—cytochrome P450. The upregulated genes in C2 were mainly enriched in Cytokine—cytokine receptor interaction, Neuroactive ligand–receptor interaction (Fig. S1E). We then performed GSEA (KEGG) enrichment on the two clusters showing that in C1 the DRUG_METABOLISM_CYTOCHROME_P450 pathway and RETINOL_METABOLISM pathway were mainly enriched, while in C2 the OOCYTE_MEIOSIS pathway and PROGESTERONE_MEDIATED_OOCYTE_MATURATION pathway were mainly enriched (Fig. 2A). These results suggest that cellular senescence is related to cell metabolism, cytokine secretion, and immune cell activation, which encourages us to further explore the effects of cellular senescence on energy metabolism, chemokine secretion, and immune cell infiltration.

### Differences in chemokine and energy metabolism-related genes among clusters

**Figure 3:**
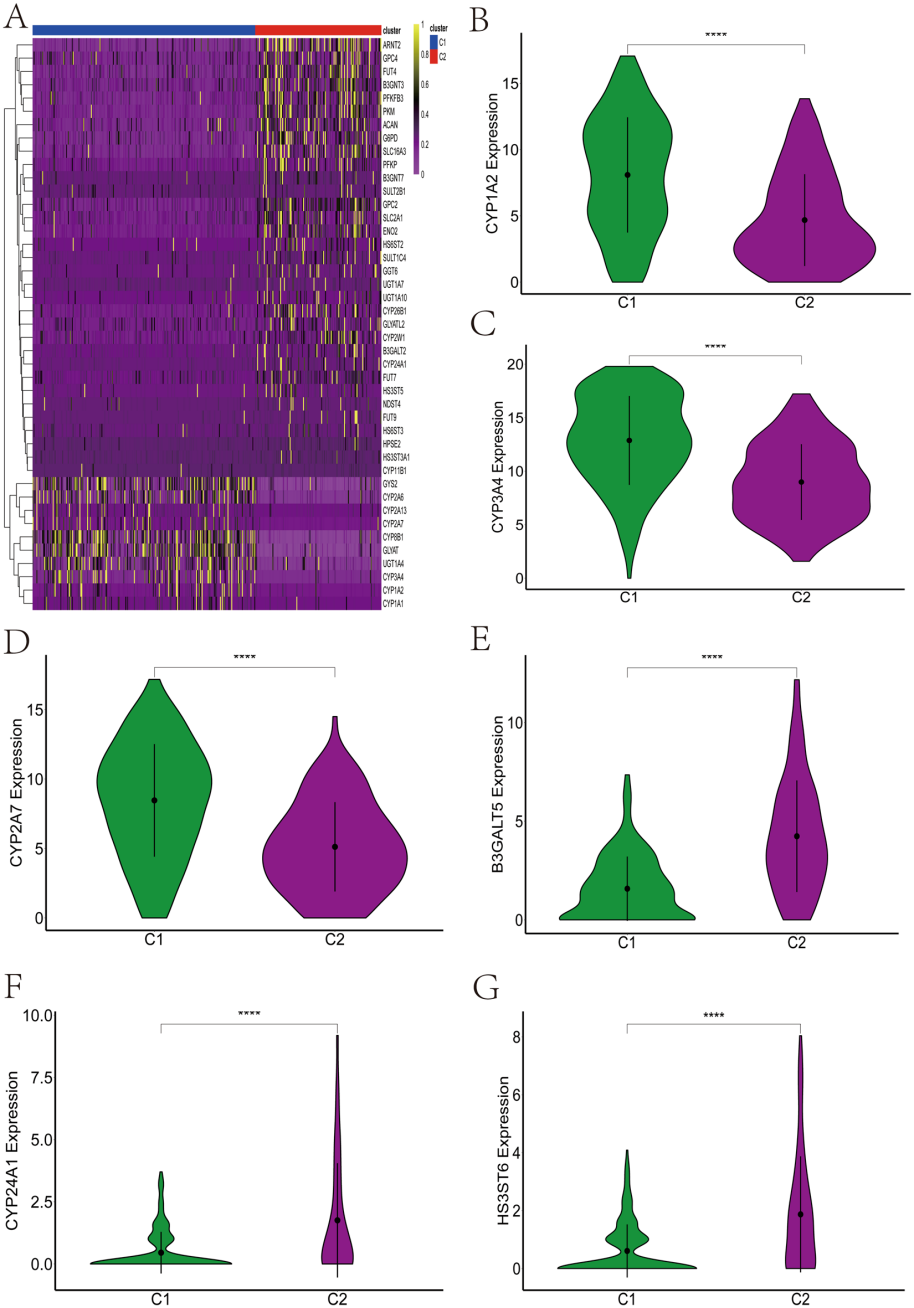
The expression levels of genes related to energy metabolism differed significantly between clusters. (**A**) Heatmap results showed that most energy metabolism-related genes were highly expressed in C2. (**B**–**D**) Energy metabolism-related genes such as CYP1A2 (**B**), CYP3A4 (**C**), CYP2A7 (**D**) were overexpressed in C1 compared to C2. (**E**–**G**) Energy metabolism related genes such as B3GALT5 (**E**), CYP24A1 (**F**), HS3ST6 (**G**) were highly expressed in C2 compared to C1.

We further compared the expression differences of chemokine and energy metabolism-related genes in C1 and C2, we found that in C2 the expression of most chemokine-related genes was significantly higher than in C1 (Fig. 2B), especially CCL26, CXCL5, CXCL6, CXCL1 (Fig. 2C–F). Similarly, we found that most of the energy metabolism-related genes were highly expressed in C2 (Fig. 3A–G). We further carried out functional enrichment analysis of the differentially expressed energy metabolism-related genes, and the results showed that these differentially expressed energy metabolism-related genes were mainly enriched in xenobiotic metabolic process, cellular response to xenobiotic stimulus, and carbohydrate biosynthetic process pathways (Fig. S2A–B). The above results further suggest that there is a link between cellular senescence and tumor microenvironment, while cellular senescence can change the energy metabolic state to some extent.

### Characterization of immune landscape in distinct cellular senescence clusters

**Figure 4:**
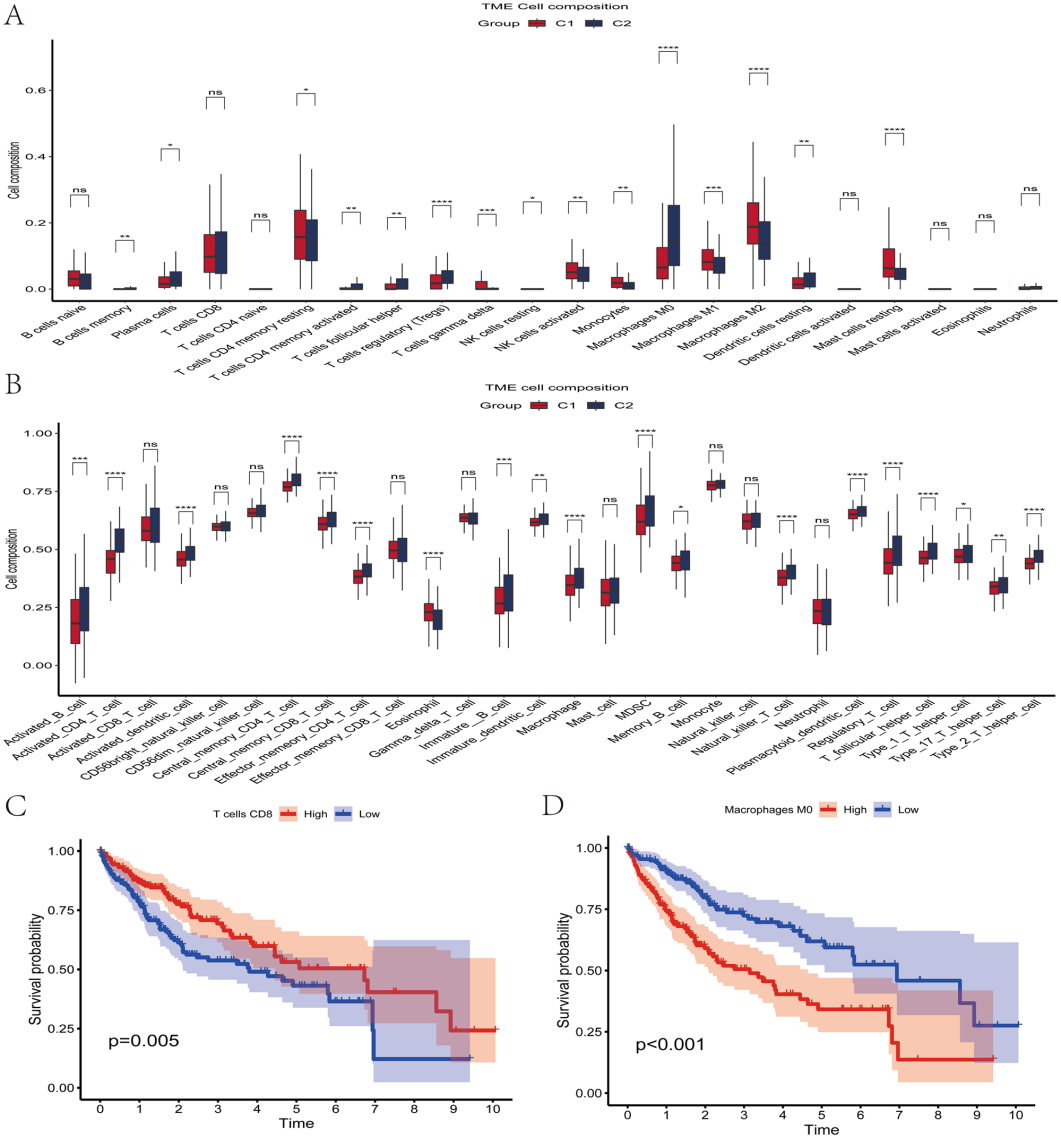

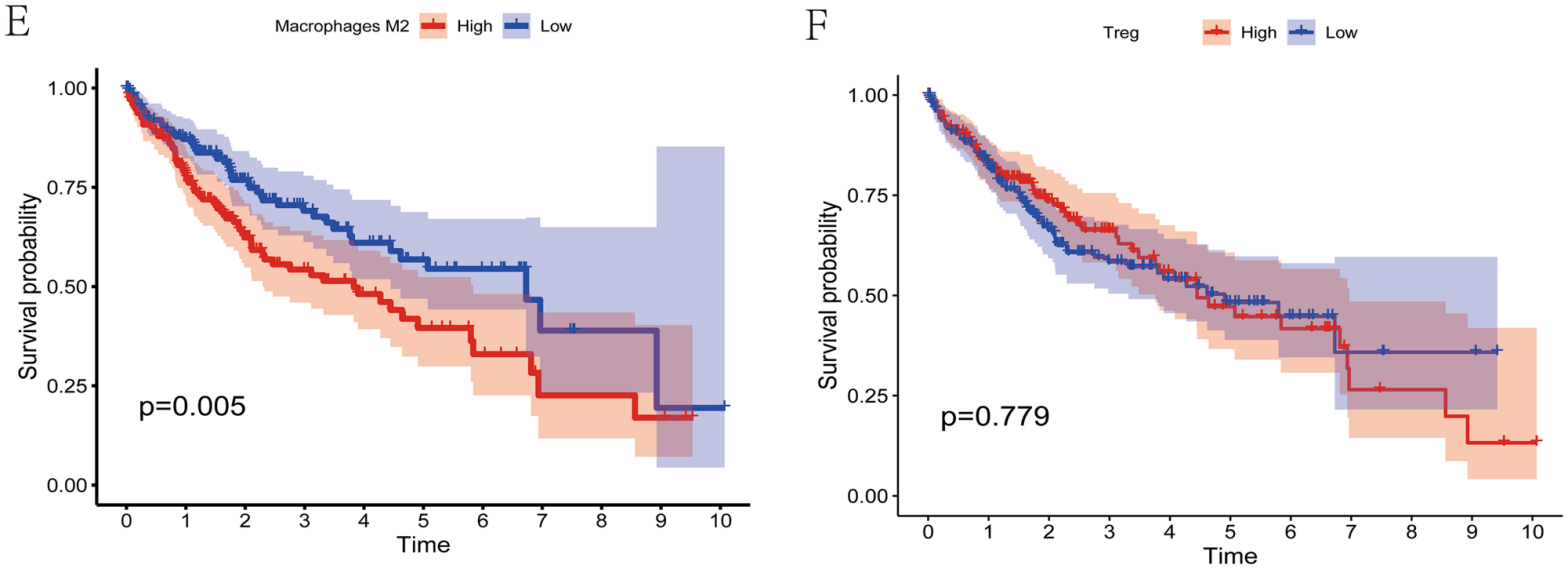
Analysis of immune cell infiltration in the tumor microenvironment. (**A**) Differences in the proportion of immune cell infiltration by tumor microenvironment species between clusters. (**B**) Differences in the proportion of 28 immune cell infiltrates in the tumor microenvironment between clusters. (**C**) Kaplan–Meier survival analysis showed that HCC patients in the highly infiltrated T cell CD8 group had longer overall survival compared to the low infiltrated T cell CD8 group. (**D**) Kaplan–Meier survival analysis showed that HCC patients in the highly infiltrated M0 Macrophages group had a shorter overall survival time compared to the low infiltrated group. (**E**) Kaplan–Meier survival analysis showed that HCC patients in the highly infiltrated M2 Macrophages group had a shorter overall survival time compared to the low infiltrated group. (**F**) Kaplan–Meier survival analysis showed no significant difference in overall survival time in the highly infiltrated Treg cell group compared to the low infiltrated group of HCC patients.

Previous studies have demonstrated a relationship between cellular senescence and tumor immune infiltration in a variety of tumor types^23,24^. We calculated the proportion of 21 immune cell species in each HCC sample using the R software CIBERSORT, while comparing the differences between immune cell components across clusters (Fig. 4A). Particularly, we found that regulatory T cells (Tregs) were markedly elevated in C2. Subsequently, we applied the ssGSEA algorithm to determine the relative ratios of 28 immune cells and immune-related pathways in each HCC sample, comparing the differences in immune cell composition between clusters (Figs. 4B, S3). We found that most immune cells were significantly enriched in C2, and interestingly, Myeloid-derived suppressor cells (MDSCs) cells (MDSCs) were significantly higher in C2 than in C1, which was concordant with previous observations linking C2 to an immunosuppressive phenotype. We further compared the effect of infiltrating immune cells on the survival of HCC patients, and we found that patients with highly infiltrated CD8T cells had a better prognosis; while highly infiltrated M0 Macrophages and M2 Macrophages cells had the opposite result (Fig. 4C–E). Interestingly, we found that the proportion of infiltrated Treg cells in the tumor microenvironment had no significant effect on the prognosis of HCC patients (*p* > 0.05) (Fig. 4F). In addition, we calculated the StromalScore, ImmuneScore, ESTIMATEScore, and TumorPurity for each HCC sample using the ESTIMATE function of R software, and the ImmuneScore of C2 was significantly higher than that of C1, in agreement with the previous results (Fig. S4A–D). Overall, these results confirm that cellular senescence is associated with the tumor microenvironment, and to some extent, contributes to the formation of an immunosuppressive microenvironment.

### Landscape of tumor mutation and prediction of immunotherapy response in different clusters

**Figure 5:**
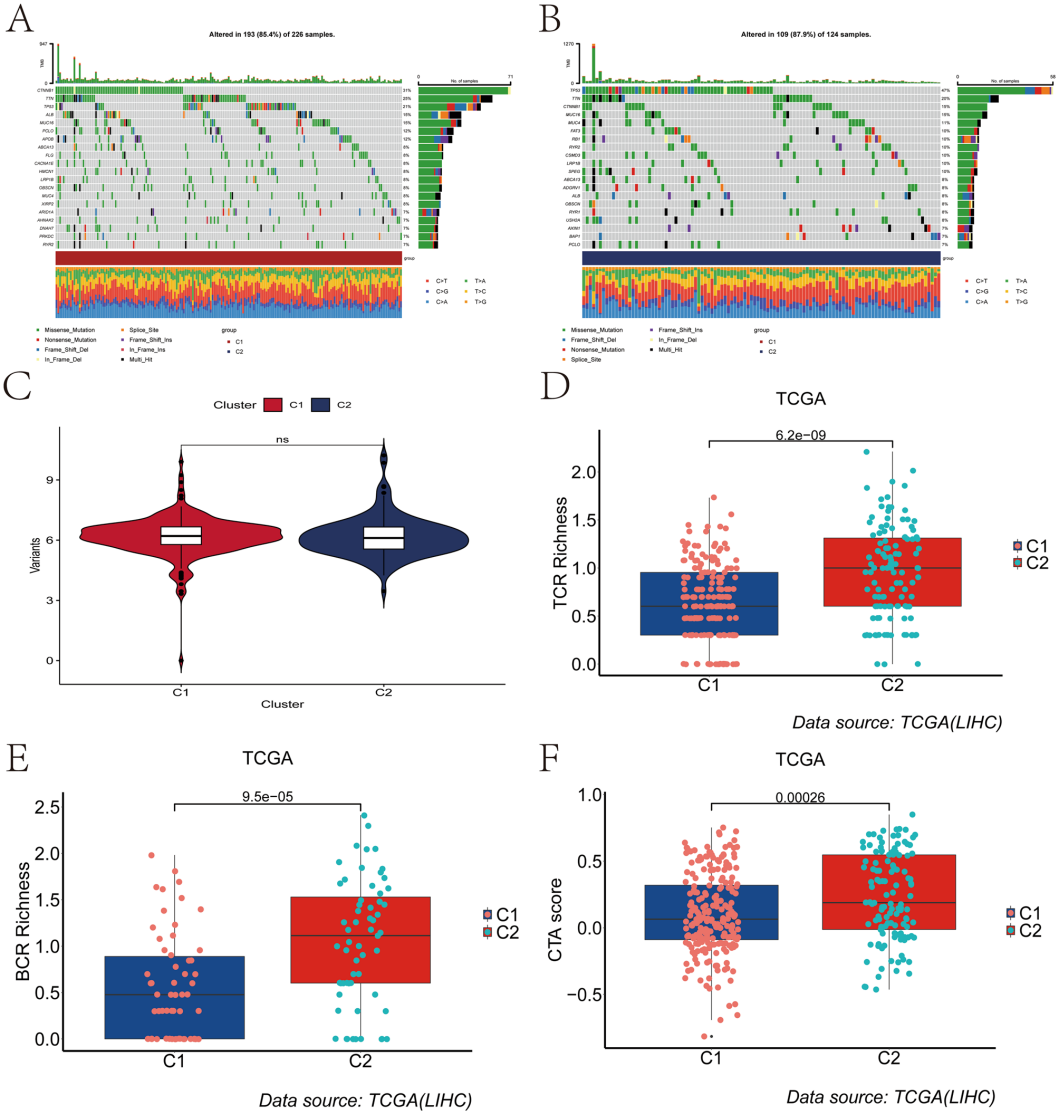
Tumor somatic gene mutation and immunotherapy response prediction. (**A**) Mutation landscape of tumor somatic cells in patients with cluster C1. (**B**) Landscape of tumor somatic cell mutations in patients with C2 clusters. (**C**) Differences in the distribution of tumor mutation burden among clusters. (**D**–**F**) Differences between different clusters in TCR Richness (**D**), BCR Richness (**E**) and CTA scores (**F**).

**Figure 6:**
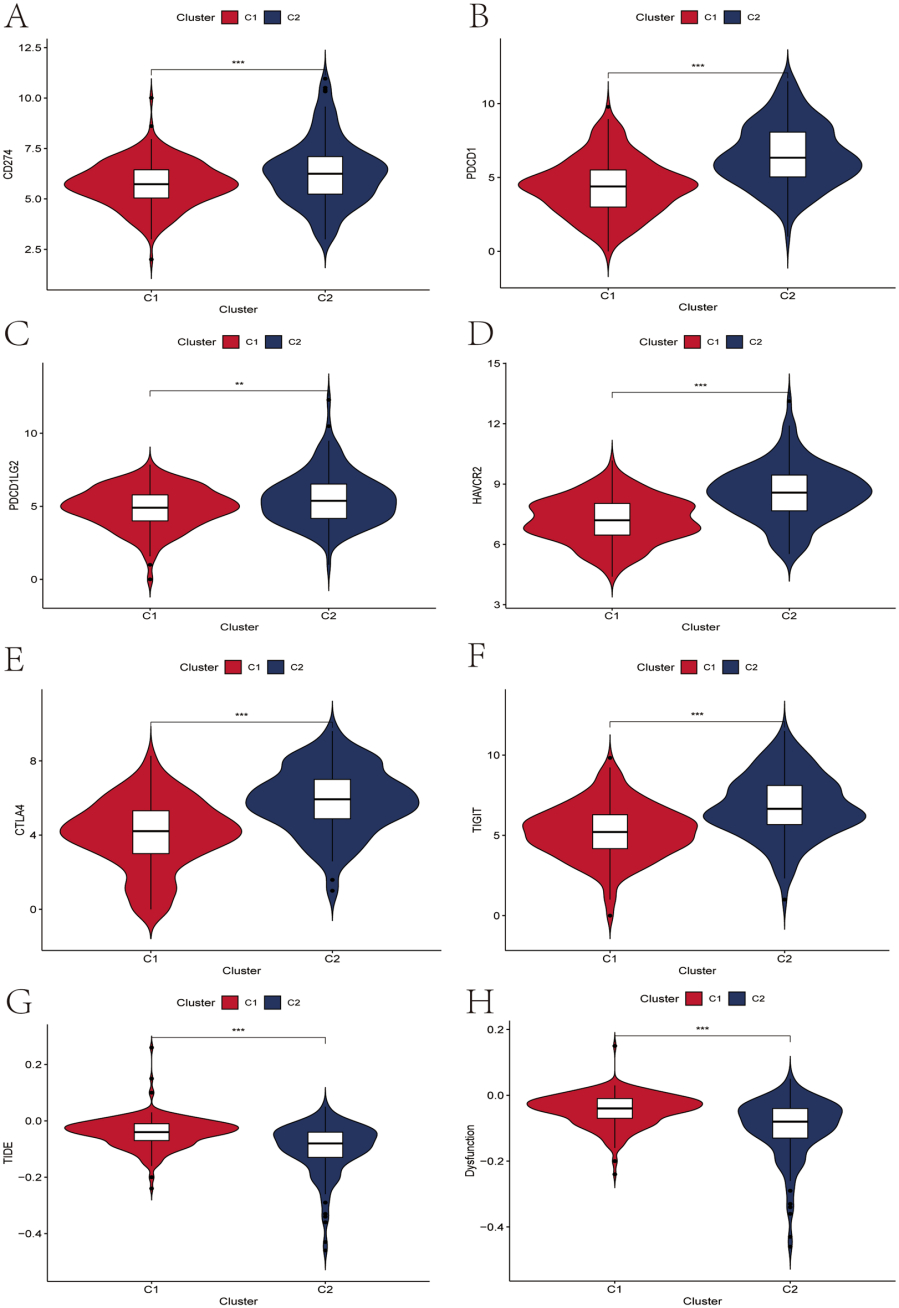
Relationship between mRNA expression levels of immune checkpoint-associated genes and different clusters. (**A**–**F**) Differences in mRNA expression levels of six immune check-related genes commonly used in HCC immunotherapy between C1 and C2, including PD-L1 (**A**), PD1 (**B**), PD-L2 (**C**), TIM-3 (**D**), CTLA4 (**E**) and TIGIT (**F**). (G-H) TIDE scores (**G**) and Dysfunction scores (**H**) were used to assess immunotherapy response.

We compared the differences in somatic mutations and tumor mutational burden (TMB) between clusters. Interestingly, C1 had the CTNNB1 gene as the major mutated gene, while C2 had the TP53 gene as the major mutated gene (Fig. 5A–B). We further compared the differences in TMB scores between different clusters, and the results showed that there was no significant difference in TMB scores between the two groups (*p* > 0.05)(Fig. 5C). MSI can be used as a marker for predictive immunotherapy in a variety of solid tumors. We further compared the MSI differences between different clusters. Interestingly, the results showed that there was no significant difference in MSI between the two clusters (*p* > 0.05), but the median MSI value of the C2 was higher than that of the C1 cluster (Fig. S5). To some extent, the C2 may be more responsive to immunotherapy than the C1. To further assess the differences in immune response between clusters, we compared the differences in TCR richness, BCR richness, and CTA scores between the two clusters. The results showed that C2 had a higher TCR richness, BCR richness, and CTA score (Fig. 5D–F), further suggesting that patients in the C2 group were more likely to respond to immunotherapy. The mRNA expression level of immune checkpoint-related genes is the basis of immunotherapy. Therefore, to further explore the complex communication between immunomodulators, immune infiltration, and cellular senescence, we explored the expression of immune checkpoint-related genes among different clusters. The results showed that the mRNA expression levels of immune checkpoint-related genes were significantly upregulated in C2 group when compared with C1 group (Fig. S6A–B). We found that immune checkpoint-related genes commonly used in liver cancer immunotherapy, including PD-L1, PD1, PD-L2, CTLA-4, TIGIT, and TIM-3, were significantly upregulated in C2 (Fig. 6A–F), further suggesting the existence of an immunosuppressive microenvironment in C2 and that immunotherapy could reverse this immunosuppressive state. Finally, we used the TIDE score to assess the clinical effectiveness of immunotherapy across clusters. In our results, C2 had the lower TIDE score and Dysfunction score (Fig. 6G–H), implying that patients in C2 could benefit more from immunotherapy than C1.

Taken together, our comprehensive analysis showed that cellular senescence clusters are significantly associated with energy metabolism, chemokines, tumor microenvironment, patient prognosis, and immunotherapy response, which may provide new insights into HCC classification system.

### Construction of the cellular senescence score for overall survival in HCC patients

**Figure 7:**
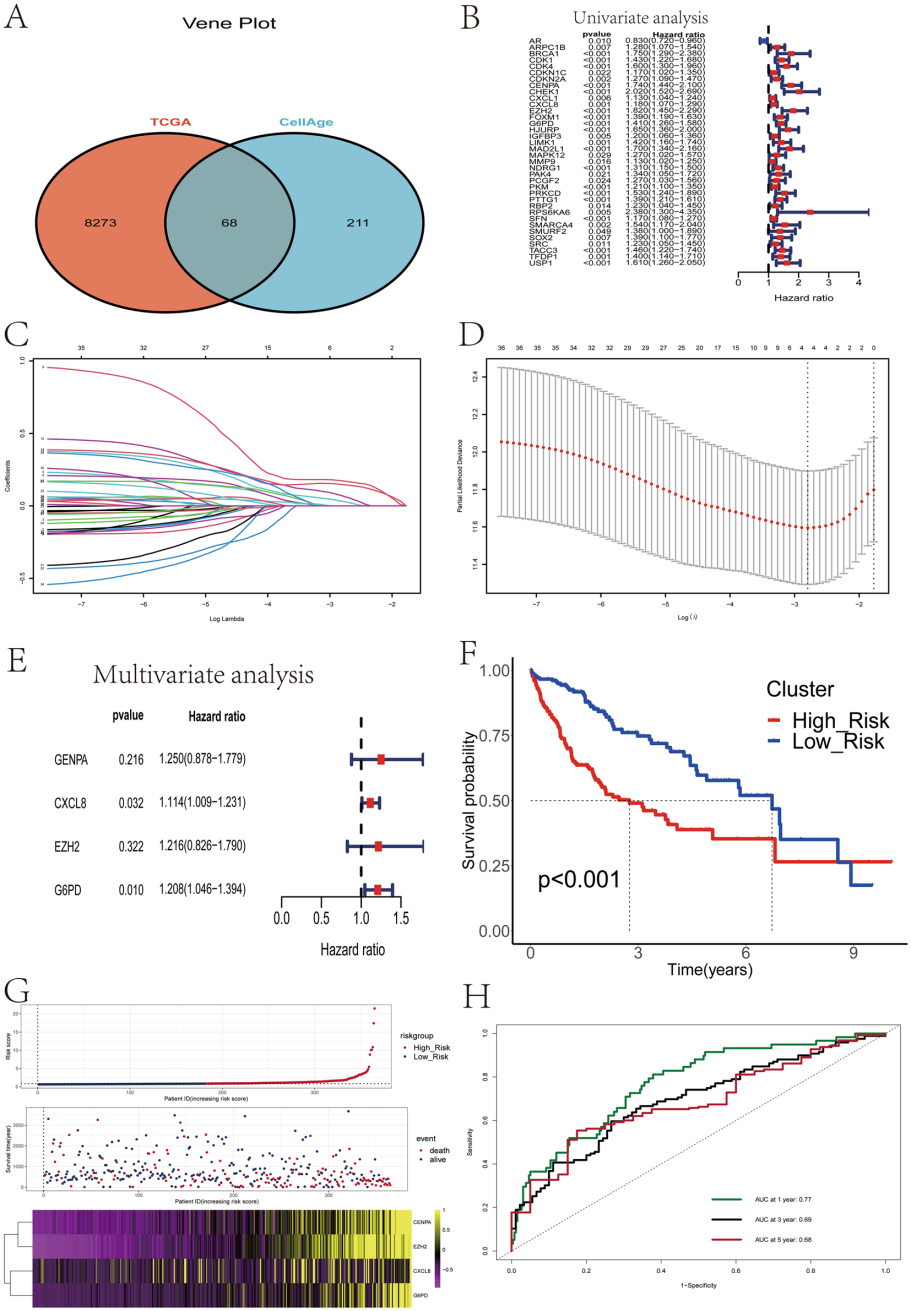
Construction and validation of a prognostic signature based on cellular senescence-related genes. (**A**) Venn diagram demonstrating the acquisition of 68 differentially expressed cellular senescence-related genes. (**B**) Results of univariate Cox regression analysis of differentially expressed cellular senescence-associated genes. (**C**) The coefficients of genes calculated by multivariate Cox regression using LASSO. (**D**) The partial likelihood deviance of genes. (**E**) Results of multivariate Cox regression analysis of four differentially expressed cellular senescence-related genes. (**F**) Kaplan–Meier curves were used to compare the overall survival of HCC patients between the high-risk and low-risk groups. (**G**) The association of risk scores with survival status and gene expression in HCC patients.(**H**) ROC curves of the prognostic signature for predicting the risk of death at 1, 3, and 5 years.

To better reflect the characteristics of C1 and C2, we constructed a risk score signature to differentiate HCC patients. First, we performed differential gene analysis for C1 and C2 with the screening criteria of *p*-value < 0.05 and logFC > 1 or logFC < -1. A total of 8341 differential genes were obtained. Subsequently, we selected 68 genes that overlapped with the set of genes associated with cellular senescence (Fig. 7A). We identified 36 prognosis-related cellular senescence genes using Cox univariate regression analysis (Fig. 7B); we then used LASSO Cox regression analysis and multifactorial Cox regression analysis to build CSS (Fig. 7C–D). Four prognostic genes (CENPA, CXCL8, EZH2, and G6PD) were identified in the training set used to construct the CSS (Fig. 7E). The risk score was calculated using the following formula: risk score = (0.22281 × CENPA gene expression) + (0.10830 × CXCL8 gene expression) + (0.19533 × EZH2 gene expression) + (0.18866 × G6PD gene expression). Patients in the training set were divided into high-risk group and low-risk group based on the median risk score. Patients in the low-risk group had significantly higher survival rates than those in the high-risk group (Fig. 7F). As shown in the heatmap, the mRNA expression levels of 4 cellular senescence-related genes were significantly higher in the high-risk group than in the low-risk group; the mortality rate of patients gradually increased with increasing risk scores (Fig. 7G). We evaluated the predictive efficacy of risk scores on the prognosis of HCC patients using ROC curves (Fig. 7H). To further verify the superiority of the CSS, we compared the CSS with other clinical features in terms of predicting the prognosis of HCC patients, and we found that the CSS ad the largest area under the curve in predicting the prognosis of HCC patients, suggesting that the CSS was significantly superior to other clinical features (Fig. S7A). We used the validation set to determine the robustness and prognostic value of CSS. To further evaluate the validity and robustness of the CSS, two independent external data sets were used to validate the CSS, and the clinical characteristics of the 2 independent datasets are presented in Supplement Table 1. We used the same calculation formula to calculate the risk scores of each HCC patient in the validation set, and divided the HCC patients in the validation set into high-risk group and low-risk group according to the median value of the risk scores. The Kaplan–Meier analysis revealed that the overall survival time of high-risk patients was considerably shorter than that of low-risk patients in two external cohorts: GSE14250 (Fig. S7B, HR = 1.687, 95% CI 1.092–2.606, *p* = 0.0185) and ICGC (Fig. S7C, HR = 2.802, 95% CI 1.458–5.387, *p* = 0.002). In two independent external validation sets, ROC curves confirmed that risk scores can effectively predict 1-, 3-, and 5-year survival in HCC patients (Fig. S7D–E).

Meanwhile, we further explored the relationship between CSS and different clusters, and we found that most patients in C2 cluster were in the high-risk group; similarly, most patients in the low-risk group belonged to C1 cluster (Fig. S7F). These results suggest that CSS can effectively respond to cellular senescence clusters. We used ROC curves to verify the mapping relationship between CSS and cellular senescence clusters, and the results showed that the CSS had better predictive efficacy for cellular senescence clusters (AUC = 0.893)(Fig. S8A). Therefore, we believe that the risk score of this signature can well reflect the characteristics of cellular senescence in HCC.

### Identification and validation of prognostic signature as an independent prognostic factor

**Figure 8:**
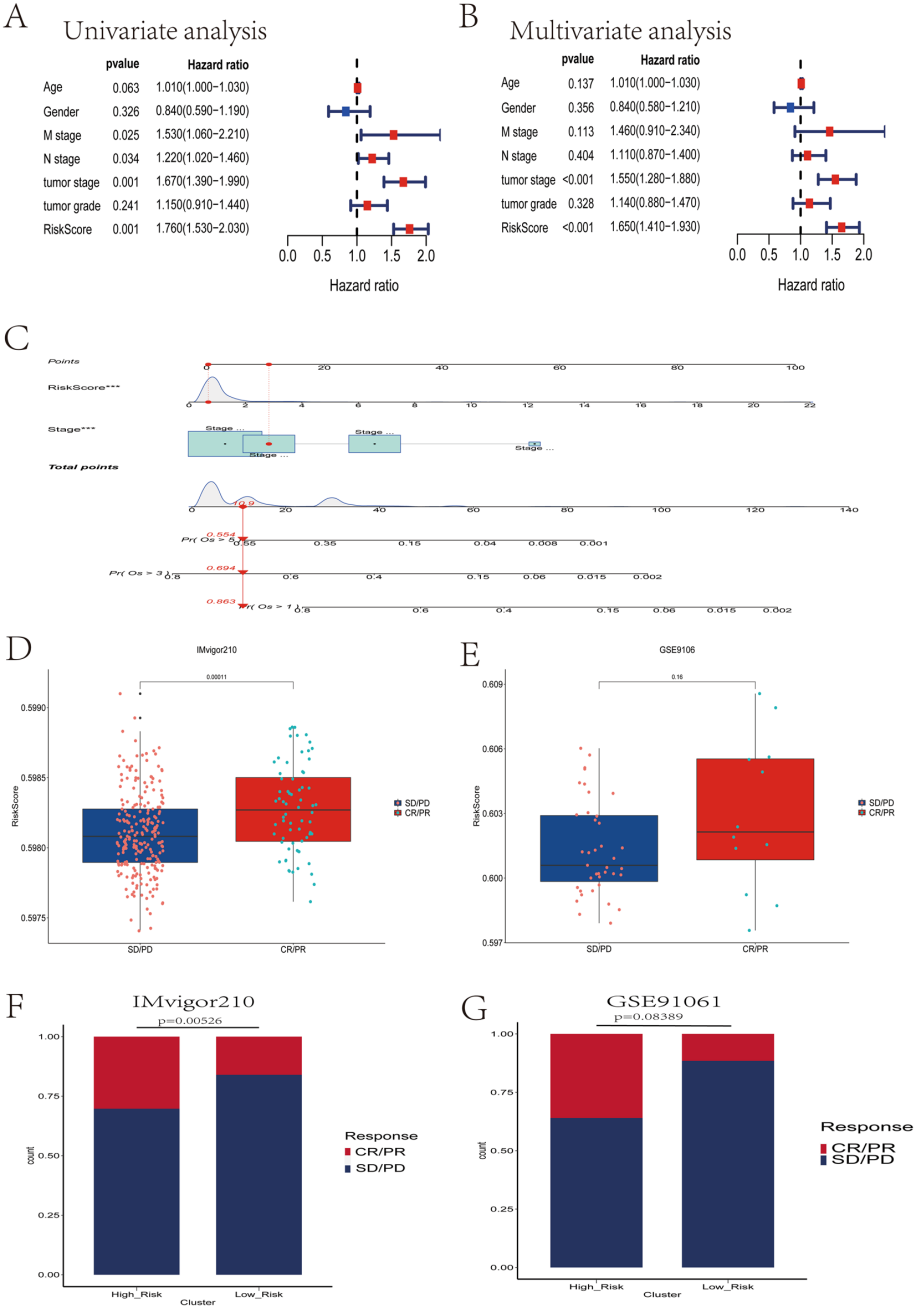
Construction of nomograms and validation of immunotherapy responses in multiple independent external datasets. (**A**) Results of univariate Cox regression analysis of risk scores and clinical characteristics. (**B**) Results of multivariate Cox regression analysis of risk scores and clinical characteristics. (**C**) Nomogram constructed from Stage and risk score. (**D**) In the IMvigor210 dataset, patients who responded to immunotherapy (CR/PR) had significantly higher risk scores than patients in the low-risk group. (**E**) In the GSE91061 dataset, patients who responded to immunotherapy (CR/PR) had a higher median risk score than patients in the low-risk group. (**F**) In the IMvigor210 dataset, the percentage of patients who responded to immunotherapy (CR/PR) was significantly higher in the high-risk group than in the low-risk group. (**G**) In the GSE91061 dataset, the percentage of patients who responded to immunotherapy (CR/PR) was higher in the high-risk group than in the low-risk group.

To further investigate the prognostic value of the prognostic signature for patients with HCC, we performed univariate Cox regression analysis and multivariate Cox regression analysis of risk scores with other clinical characteristics. The results suggested that Stage and risk score were independent risk factors affecting HCC patients (Fig. 8A–B). To better predict the probability of survival in HCC patients, we created a predictive nomogram based on the integration of risk scores and Stage (Fig. 8C). ROC curves confirm that nomogram can effectively predict the survival of HCC patients at 1, 3, and 5 years (Fig. S8B). Calibration curves further confirm that nomogram can effectively predict the overall survival time of HCC patients (Fig. S8C). This indicates high accuracy of our nomograms. In addition, decision curves confirmed that nomograms constructed based on risk scores and Stage significantly outperformed nomograms constructed on other clinical characteristics (Fig. S8D).

### Independent external immunotherapy data validate the prediction of immunotherapy response

To further validate the predictive value of the CSS for immunotherapy response, we downloaded two independent external immunotherapy data for a comprehensive analysis (IMvigor210, GSE9106). We calculated risk scores for patients receiving immunotherapy in both immunotherapy datasets using the same formula as in the training set, and divided patients receiving immunotherapy into high-risk and low-risk groups based on the median value of the risk scores. We found a significant correlation between risk score and immunotherapy response (Fig. 8D–E), with a significantly higher proportion of CR/PR patients in the high-risk patients than in the low-risk group in the two independent external immunotherapy data (Fig. 8F–G). These validation results all confirm that patients in the high-risk group may be more responsive to immunotherapy and more suitable for immunotherapy. Thus, our novel CSS can effectively predict the response to immunotherapy in HCC patients.

### qRT-PCR confirms overexpression of four cellular senescence-related genes and two chemokine-related genes in HCC tissues

**Figure 9:**
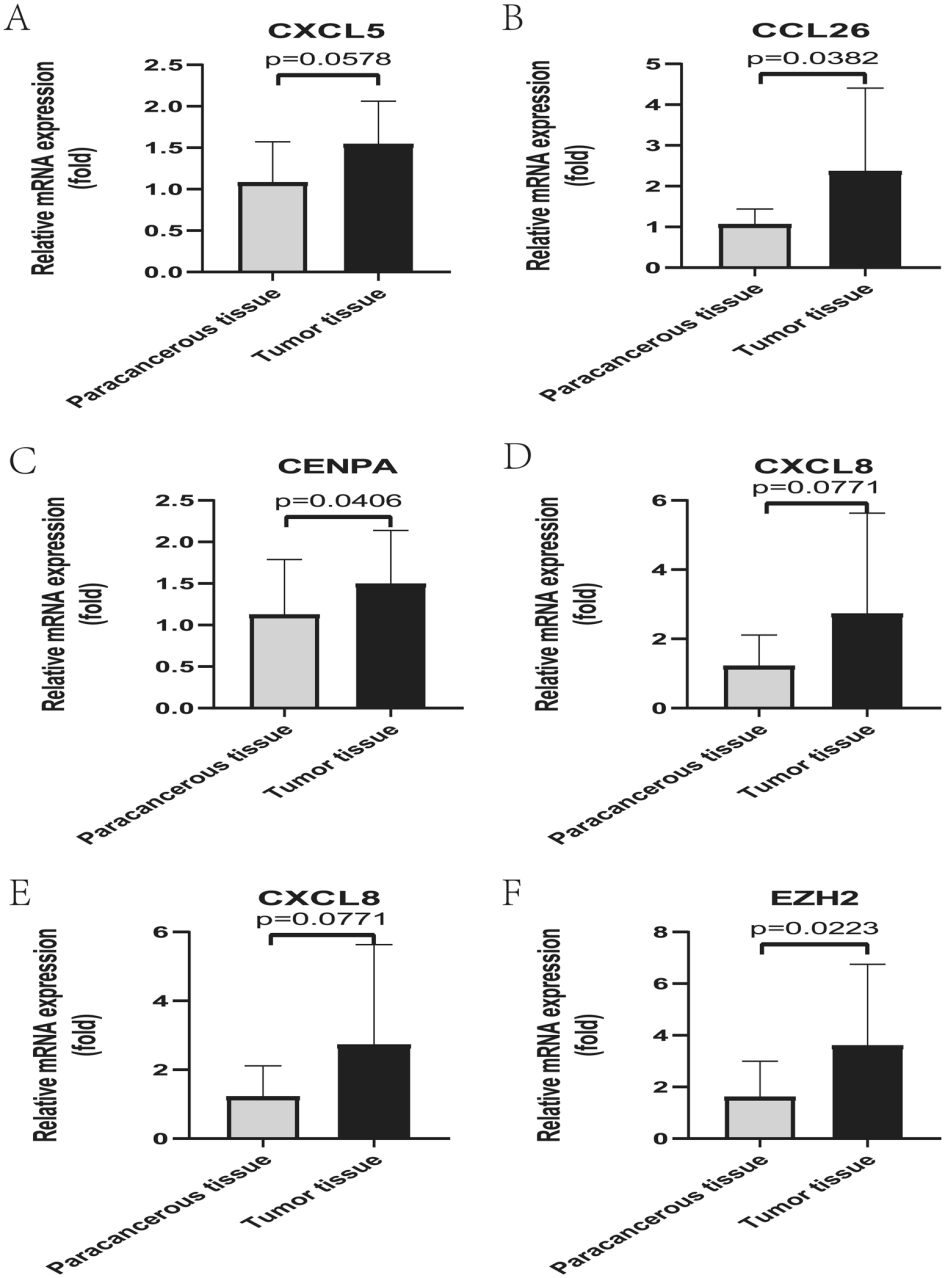
The mRNA expression of four cellular senescence-related genes and two chemokine-related genes in HCC tissues was confirmed by qRT-PCR. (**A**–**B**) The qRT-PCR results confirmed for 2 chemokine-related genes overexpressed in HCC tissues, including CXCL5 (**A**), CXCL26 (**B**). (**C**–**F**) The qRT-PCR results confirmed that four cellular senescence-associated genes used to construct the prognostic model were overexpressed in HCC tissues, including CENPA (**C**), CXCL8 (**D**), EZH2 (**E**), and G6PD (**F**).

To validate the robustness of the CSS, we collected tumor tissue samples and paraneoplastic tissue samples from 12 patients with HCC confirmed by postoperative pathological pathology from the clinic and verified the mRNA expression levels of four cellular senescence-related genes and two chemokine-related genes using qRT-PCR. The qRT-PCR results demonstrated that four cellular senescence-related genes (CENPA, CXCL8, EZH2, and G6PD) and two chemokine-related genes (CCL26 and CXCL5) were overexpressed in tumor tissues from HCC patients compared with paraneoplastic tissues (Fig. 9). These results suggest that our novel prognostic signature constructed based on cellular senescence-related genes is highly robust, and this robustness was validated in clinical samples.

## Discussion

HCC is the sixth most common cancer in the world, and it is the fourth most lethal^25^. The introduction of immune checkpoint inhibitors in recent years has dramatically changed the treatment of hematologic and solid tumors, including HCC^26,27^. However, the immunotherapy of HCC has proven a challenge for the era of personalized therapy due to inter- and intra-tumoral heterogeneity. At the same time, significant problems such as low response rates to immunotherapy still exist in clinical practice, so it is crucial to use biomarkers to predict prognosis and immunotherapy response, and thus to implement personalized therapy for individuals.

Senescence is an injury-induced stress program that activates a series of pleiotropic cytokines, chemokines, growth factors, and proteases of the senescence-associated secretory phenotype (SASP), ultimately leading to persistent suppression of tumor cells and remodeling of the tumor immune microenvironment^28^. A growing number of studies have confirmed that senescent cells can be removed by SASP to stimulate the immune response and thus achieve clearance^23^. Thus, SASPs are positive for the body in the short term. However, in the long term, these functions could become detrimental in the context of immunosuppression of cancer to promote tumor development^15,29,30^. The association and value between cellular senescence, energy metabolism, chemokines, and tumor immune infiltration have not been reported, especially in HCC. Therefore, studies on the biological mechanisms and prognostic biomarkers of HCC concerning cellular senescence-related genes may offer an opportunity to identify HCC subtypes, improving the future application of precision focused treatments for HCC. In addition, the existing immunotherapeutic drugs suffer from both low response rate to immunotherapy and drug tolerance, and failed to meet clinical needs consequently. So revealing the immune microenvironment from the level of cellular senescence will provide new perspectives for the development of related drugs.

With the development of second-generation sequencing technologies, transcriptome analysis has paved the way for the identification of new prognostic and predictive biomarkers to discern the heterogeneity and complexity of tumors to develop new, individualized treatment strategies. Based on the expression of cellular senescence-related genes, we randomly divided the 365 HCC samples in the TCGA database into C1 and C2. Interestingly, we found significant differences in immune cell infiltration, energy metabolism, and chemokines between C1 and C2 The findings suggest that cellular senescence-related genes may contribute to different biological processes and immune phenotypes, while cellular senescence-related genes are somehow intrinsically linked to the expression of energy metabolism and chemokine-related genes. These genes have been associated with different tumorigenesis and anti-cancer immunity of individual tumors. Cellular senescence C2 is significantly enriched in cell division, and tumor-related signaling pathways, including Oocyte meiosis, Cell cycle; also associated with cellular interactions, including Neuroactive ligand-receptor interaction, Cytokine-cytokine receptor interaction. Interestingly, immune infiltrating cell analysis showed a higher percentage of MDSC, and Tregs cells in C2, suggesting that patients in C2 may be associated with an immunosuppressed state. Accordingly, immune checkpoint-related genes were significantly more highly expressed in C2, further confirming the existence of an immunosuppressive microenvironment in C2. Also, we found a lower TIDE score in C2, suggesting that patients in C2 are more likely to benefit from immunotherapy. Taken together, the newly identified cellular senescence clusters might provide novel insights into classification system of HCC.

We constructed a novel prognostic signature based on the expression of four cellular senescence-related genes (CENPA, IL8, EZH2, G6PD) by the stochastic survival forest method. In addition, we merged prognostic signatures with clinical characteristic factors and constructed Nomograms as a way to quantify risk scores and survival probabilities. Compared with other traditional predictors, the prognostic signature based on cellular senescence-related genes showed better accuracy and discriminatory performance in survival prediction. Notably, our results imply that CSS is an independent prognostic factor for HCC patients and also a predictor of immunotherapy. It has been suggested that a high-risk score may be associated with a favorable response to ICI treatment. Risk scores coupled with specific immune checkpoints may serve as predictive biomarkers of ICI response and prognosis.

All four related genes of our cellular senescence-related gene signature have been demonstrated in a variety of tumors. For example, a report by Li et al. found that CENPA overexpression was associated with advanced histological grade, positive serum HBsAg status, Ki-67 expression, and p53 immunopositivity^31^. Shabangu's study showed that persistent high levels of HCV can induce altered cell adhesion and migration-promoting gene expression through the CXCL8-SRC signaling pathway^32^. A study by Bae et al.^33^. concluded that overexpression of EZH2 was significantly associated with poorer prognosis in HCC patients and that EZH2 could be a potential target for HCC immunotherapy. Lu et al.^34^. found that G6PD induces epithelial-mesenchymal transition through activation of signal transducer and activator of transcription 3 (STAT3) pathway, which contributes to HCC migration and invasion of hepatocellular carcinoma cells.

Overall, our study provides important implications for clinical research. First, we have developed and discovered a novel scoring system that can classify patients with different treatment strategies based on different risk subgroups. We found that the risk score correlated with immune cell infiltration in TME, with more immune cell infiltration in the high-risk group, but also a higher proportion of immunosuppressive cells such as MDSC, and Tregs cells, suggesting an immunosuppressive state in HCC patients with high-risk scores. In addition, we found that HCC patients in the high-risk group exhibited lower TIDE scores and higher expression of immune checkpoint-related genes, suggesting a possible higher response to immunotherapy. Our signature showed better predictive power compared to other existing biological markers. These results suggest that CSS may further stratify patient response to immunotherapy in HCC. Thus, our study of the effect of cellular senescence on TME may enhance the understanding of immunotherapy response heterogeneity. Second, we found that cellular senescence correlated with the expression of energy metabolism and chemokine-related genes, with higher expression of energy metabolism and chemokine-related genes in HCC patients with higher risk scores, suggesting more vigorous energy metabolism and stronger inflammatory response in senescent cells. Glycolysis is a feature of both cancer cell progression and T cell activation^35^. Previous reports have suggested that microenvironmental glucose competition may be a driver of immunosuppression by depleting necessities required to maintain effector T cell function, whereas blockade of glycolysis in malignant cells can boost the efficiency of immunotherapy and metabolic remodeling between tumor cells and T cells^36^. Therefore, reflecting the cellular senescence status from the perspective of tumor energy metabolism and inflammatory response will help us to have a deeper understanding of tumor cell senescence. Taken together, uncovering the relationship between tumor cell senescence and energy metabolism, TME, and immunotherapy responses may provide a perspective on cellular senescence from an immune, metabolic, and inflammatory perspective, allowing us to discover how to effectively reshape the immunosuppressive microenvironment by inhibiting the senescence process or destroying senescent cells, so-called anti-aging therapies.

Although our study has value in immunotherapy and prognosis for HCC, there are certain flaws in this study. First, the four cellular senescence-related gene sets we constructed are based on public databases, and therefore, external multicenter samples are needed for validation. Second, prospective clinical trials are necessary to verify the applicability of our findings in patients with HCC receiving immunotherapy. Third, the regulatory mechanism of cellular senescence-related genes on TME needs to be further confirmed in vitro and in vivo experiments. Finally, the preliminary interpretation of mechanisms underlying the association between cellular senescence-related genes and worse response to ICIs must be further elucidated using basic experiments.

## Conclusions

Finally, the cellular senescence risk profile presented in this study can be used to predict survival, identify molecular subtypes of HCC that may respond to immunotherapy, and provide a basis for clinical treatment planning. In the future, this signature could provide a reliable predictive tool for HCC patients.

## Supplementary Information

Supplementary Legends.

Supplementary Figure S1.

Supplementary Figure S2.

Supplementary Figure S3.

Supplementary Figure S4.

Supplementary Figure S5.

Supplementary Figure S6.

Supplementary Figure S7.

Supplementary Figure S8.

Supplementary Tables.

## Data Availability

The 279 cellular senescence-associated genes were downloaded from the CellAge database (https://genomics.senescence.info/cells/). The datasets generated and analyzed during the current study are available in the https://portal.gdc.cancer.gov/ and https://dcc.icgc.org/projects/LIRI-JP.

## References

[1] Sung H, Ferlay J, Siegel RL (2021). Global cancer statistics 2020: GLOBOCAN estimates of incidence and mortality worldwide for 36 cancers in 185 countries. CA Cancer J. Clin..

[2] Llovet JM, Kelley RK, Villanueva A (2021). Hepatocellular carcinoma. Nat. Rev. Dis. Primers.

[3] Llovet JM, De Baere T, Kulik L (2021). Locoregional therapies in the era of molecular and immune treatments for hepatocellular carcinoma. Nat. Rev. Gastroenterol. Hepatol..

[4] Llovet JM, Montal R, Sia D (2018). Molecular therapies and precision medicine for hepatocellular carcinoma. Nat. Rev. Clin. Oncol..

[5] Ling Y, Liu J, Qian J (2020). Recent advances in multi-target drugs targeting protein kinases and histone deacetylases in cancer therapy. Curr. Med. Chem..

[6] Zhang J, Dang F, Ren J (2018). Biochemical aspects of PD-L1 regulation in cancer immunotherapy. Trends Biochem. Sci..

[7] Finn RS, Qin S, Ikeda M (2020). Atezolizumab plus bevacizumab in unresectable hepatocellular carcinoma. N. Engl. J. Med..

[8] Champiat S, Ferrara R, Massard C (2018). Hyperprogressive disease: Recognizing a novel pattern to improve patient management. Nat. Rev. Clin. Oncol..

[9] Scheiner B, Kirstein MM, Hucke F (2019). Programmed cell death protein-1 (PD-1)-targeted immunotherapy in advanced hepatocellular carcinoma: Efficacy and safety data from an international multicentre real-world cohort. Aliment. Pharmacol. Ther..

[10] Gorgoulis V, Adams PD, Alimonti A (2019). Cellular senescence: Defining a path forward. Cell.

[11] Campisi J (2013). Aging, cellular senescence, and cancer. Annu. Rev. Physiol..

[12] Hernandez-Segura A, Nehme J, Demaria M (2018). Hallmarks of cellular senescence. Trends Cell Biol..

[13] Demaria M, O'Leary MN, Chang J (2017). Cellular senescence promotes adverse effects of chemotherapy and cancer relapse. Cancer Discov..

[14] Lasry A, Ben-Neriah Y (2015). Senescence-associated inflammatory responses: Aging and cancer perspectives. Trends Immunol..

[15] Coppé JP, Desprez PY, Krtolica A (2010). The senescence-associated secretory phenotype: The dark side of tumor suppression. Annu. Rev. Pathol..

[16] Liu X, Hartman CL, Li L (2021). Reprogramming lipid metabolism prevents effector T cell senescence and enhances tumor immunotherapy. Sci. Transl. Med..

[17] Choi YW, Kim YH, Oh SY (2021). Senescent tumor cells build a cytokine shield in colorectal cancer. Adv. Sci. (Weinh.).

[18] Westdorp H, Fennemann FL, Weren RD (2016). Opportunities for immunotherapy in microsatellite instable colorectal cancer. Cancer Immunol. Immunother.

[19] Lorenzi M, Amonkar M, Zhang J (2020). Epidemiology of Microsatellite Instability High (MSI-H) and Deficient Mismatch Repair (dMMR) in solid tumors: A structured literature review. J. Oncol..

[20] Kloor M, von Knebel DM (2016). The immune biology of microsatellite-unstable cancer. Trends Cancer.

[21] Hause RJ, Pritchard CC, Shendure J (2016). Classification and characterization of microsatellite instability across 18 cancer types. Nat. Med..

[22] Huang TX, Fu L (2019). The immune landscape of esophageal cancer. Cancer Commun. (Lond.).

[23] Schneider JL, Rowe JH, Garcia-de-Alba C (2021). The aging lung: Physiology, disease, and immunity. Cell.

[24] Eggert T, Wolter K, Ji J (2016). Distinct functions of senescence-associated immune responses in liver tumor surveillance and tumor progression. Cancer Cell.

[25] Hwang YJ, Lee Y, Park H (2021). Prognostic significance of viable tumor size measurement in hepatocellular carcinomas after preoperative locoregional treatment. J. Pathol. Transl. Med..

[26] Ingles GA, Au L, Mason R (2019). Building on the anti-PD1/PD-L1 backbone: Combination immunotherapy for cancer. Expert Opin. Investig. Drugs.

[27] Finkelmeier F, Waidmann O, Trojan J (2018). Nivolumab for the treatment of hepatocellular carcinoma. Expert Rev. Anticancer Ther..

[28] Chibaya L, Snyder J, Ruscetti M (2022). Senescence and the tumor-immune landscape: Implications for cancer immunotherapy. Semin. Cancer Biol..

[29] Lopes-Paciencia S, Saint-Germain E, Rowell MC (2019). The senescence-associated secretory phenotype and its regulation. Cytokine.

[30] Basisty N, Kale A, Jeon OH (2020). A proteomic atlas of senescence-associated secretomes for aging biomarker development. PLoS Biol.

[31] Li Y, Zhu Z, Zhang S (2011). ShRNA-targeted centromere protein A inhibits hepatocellular carcinoma growth. PLoS ONE.

[32] Shabangu CS, Siphepho PY, Li CY (2021). The persistence of hepatitis C virus infection in hepatocytes promotes hepatocellular carcinoma progression by pro-inflammatory interluekin-8 expression. Biomedicines.

[33] Bae AN, Jung SJ, Lee JH (2022). Clinical value of EZH2 in hepatocellular carcinoma and its potential for target therapy. Medicina (Kaunas).

[34] Lu M, Lu L, Dong Q (2018). Elevated G6PD expression contributes to migration and invasion of hepatocellular carcinoma cells by inducing epithelial-mesenchymal transition. Acta Biochim. Biophys. Sin. (Shanghai).

[35] Sugiura A, Rathmell JC (2018). Metabolic barriers to T cell function in tumors. J. Immunol..

[36] Lim AR, Rathmell WK, Rathmell JC (2020). The tumor microenvironment as a metabolic barrier to effector T cells and immunotherapy. Elife.

