# Supplementary material for: Cellular senescence affects energy metabolism, immune infiltration and immunotherapeutic response in hepatocellular carcinoma
Source: Sci Rep. 2023 Jan 20;13:1137. doi: 10.1038/s41598-023-28436-z (PMC9860043; doi:10.1038/s41598-023-28436-z)
Supplement: Supplementary file 1 — Supplementary Legends. [file 41598_2023_28436_MOESM1_ESM.docx]

**Figure legends**

**Figure S1** Molecular mechanisms and signaling pathways behind the different clusters. (A) The volcano map shows the genes that are highly expressed in different clusters. (B) Heatmap showing genes differentially expressed in C1 and C2. (C) Results of GO functional enrichment analysis of genes highly expressed in C1. (D) (C) Results of GO functional enrichment analysis of genes highly expressed in C2. (E) Results of KEGG functional enrichment analysis of genes differentially expressed.

**Figure S2** Pathway enrichment of differentially expressed genes related to energy metabolism. (A) Results of GO functional enrichment analysis of differentially expressed energy metabolism-related genes. (B) Results of KEGG functional enrichment analysis of differentially expressed energy metabolism-related genes.

**Figure S3** Heatmap shows the difference in the proportion of 28 types of immune cells between C1 and C2.

**Figure S4** Differences in immune infiltration scores between C1 and C2;(A-D) Differences between C1 and C2 in ImmuneScore(A), StromalScore(B), TumorPurity(C) and ESTIMATEScore(D).

**Figure S5** Differences in MSI scores between C1 and C2.

**Figure S6** Differences in the expression levels of immune checkpoint-related genes between C1 and C2. (A) Most immune checkpoint-related genes are highly expressed in C2. (B) Heatmaps showed that most immune checkpoint related genes were highly expressed in C2 compared to C1.

**Figure S7** Multiple independent external datasets to validate the predictive performance of CSS. (A) ROC curves show higher predictive power of CSS for prognosis of HCC patients compared to other clinical features. (B) In the GSE14520 dataset, overall survival time was significantly worse for patients in the high-risk group than for those in the low-risk group. (C) In the ICGC dataset, overall survival time was significantly worse for patients in the high-risk group than for those in the low-risk group. (D) In the GSE14520 data set, CSS showed good predictive performance for the survival of HCC patients 1, 3 and 5 years. (E) In the ICGC data set, CSS showed good predictive performance for the survival of HCC patients 1, 3 and 5 years. (F) C2 has a strong correspondence with the high-risk group, while C1 has a strong correspondence with the low-risk group.

**Figure S8** Validation of the predictive efficacy of nomogram for the prognosis of HCC patients. (A) ROC curves show good predictive efficacy of CSS for patients with C2. (B) ROC curves show that CSS has good predictive efficacy for 1, 3 and 5 year survival in HCC patients. (C) Calibration curves show strong predictive power of nomogram for 1-, 3-, and 5-year survival in HCC patients. (D) Decision curves show that the nomogram model outperforms other models.
