# Supplementary figures and images for: Cellular senescence affects energy metabolism, immune infiltration and immunotherapeutic response in hepatocellular carcinoma
Source: Sci Rep. 2023 Jan 20;13:1137. doi: 10.1038/s41598-023-28436-z (PMC9860043; doi:10.1038/s41598-023-28436-z)

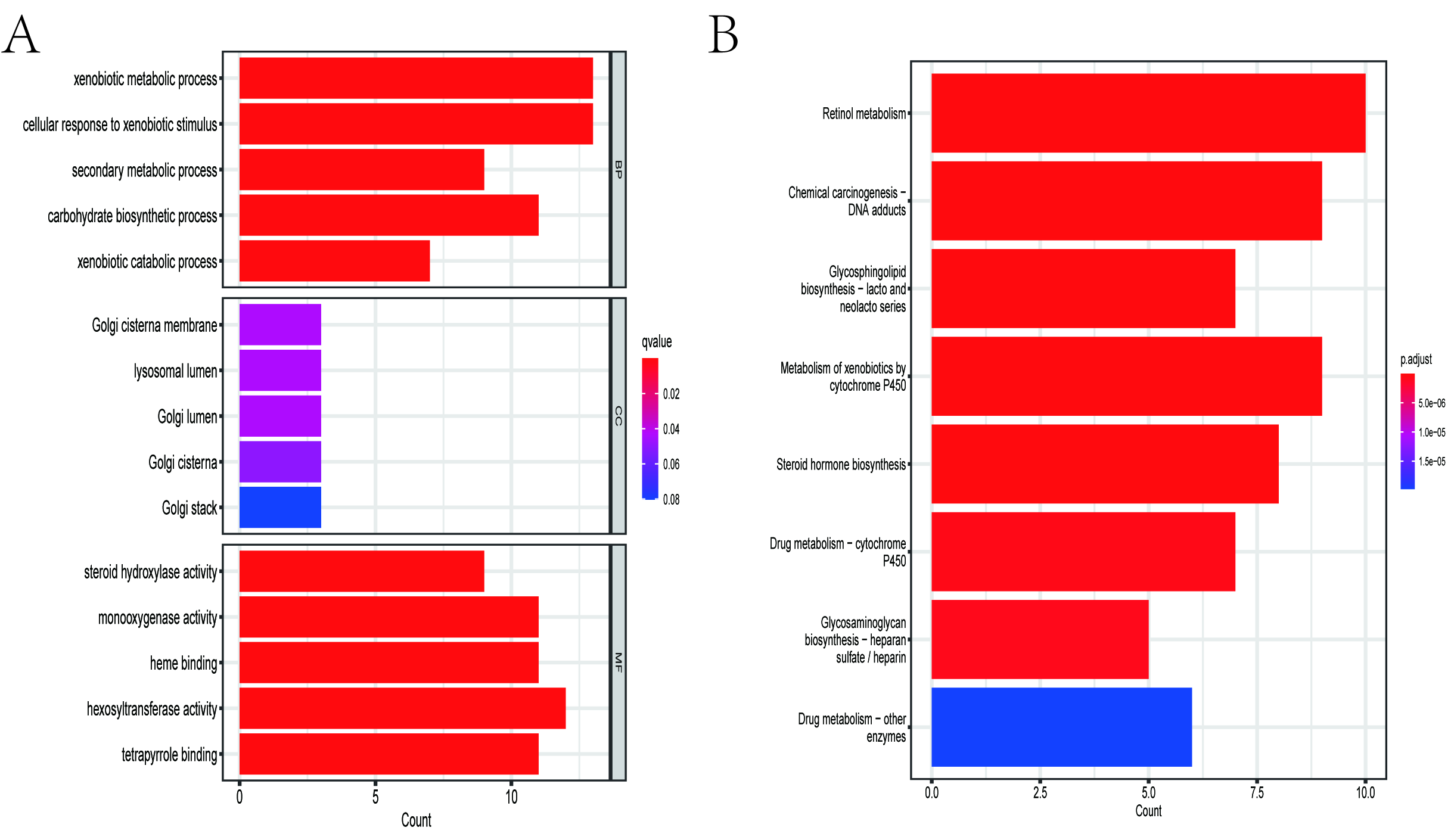

Supplement: Supplementary file 3 — Supplementary Figure S2. [file 41598_2023_28436_MOESM3_ESM.tif]

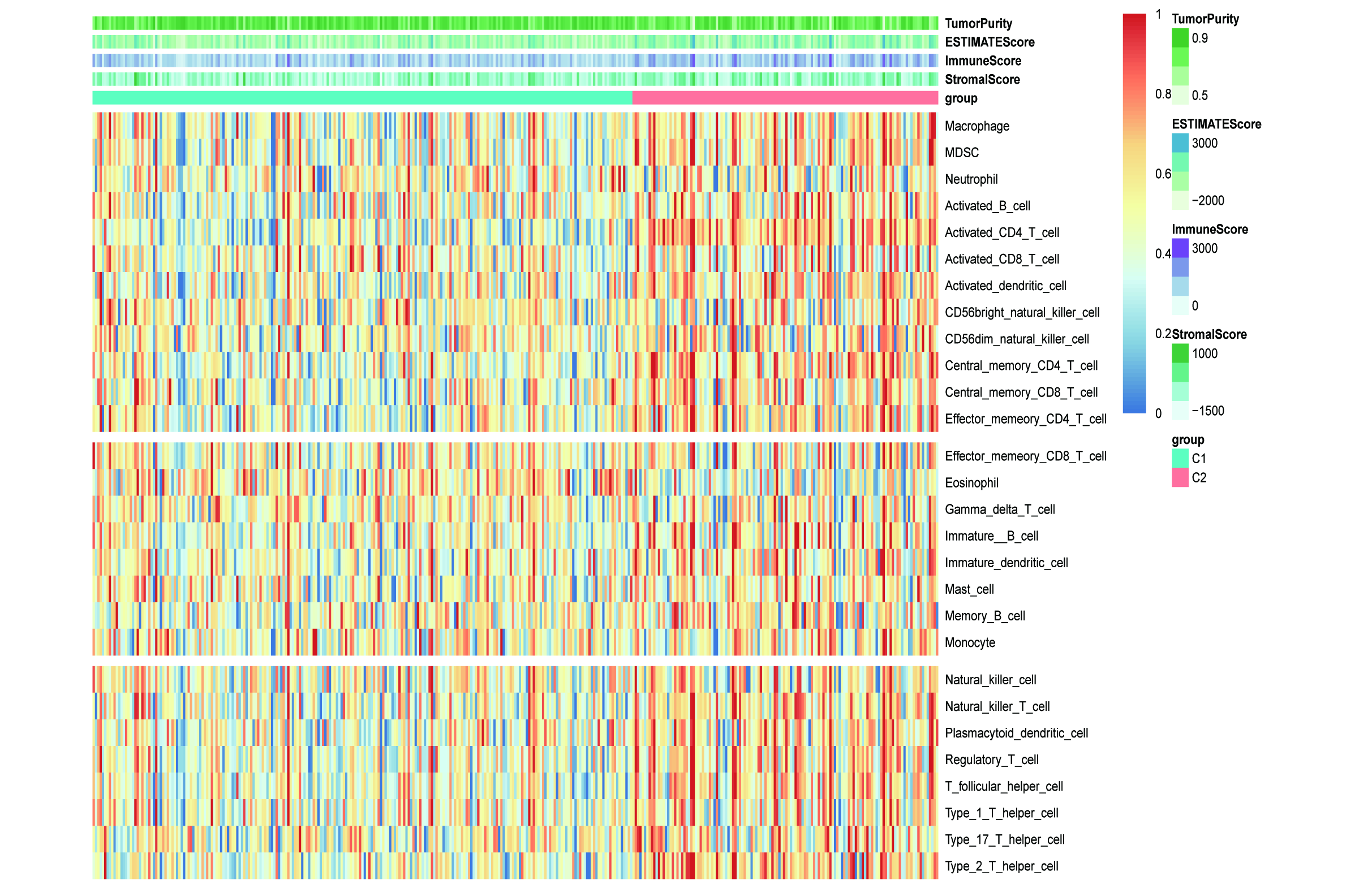

Supplement: Supplementary file 4 — Supplementary Figure S3. [file 41598_2023_28436_MOESM4_ESM.tif]

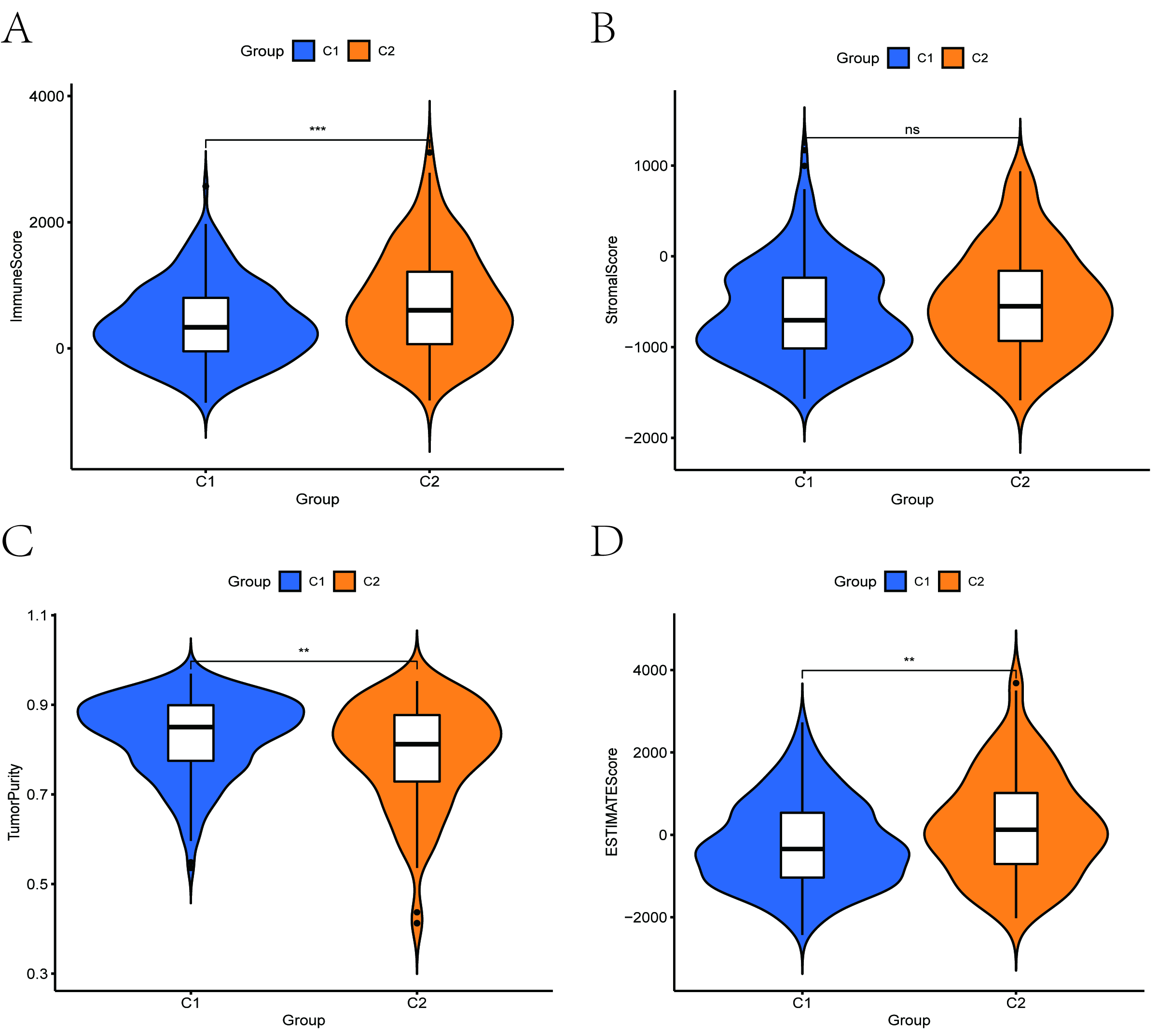

Supplement: Supplementary file 5 — Supplementary Figure S4. [file 41598_2023_28436_MOESM5_ESM.tif]

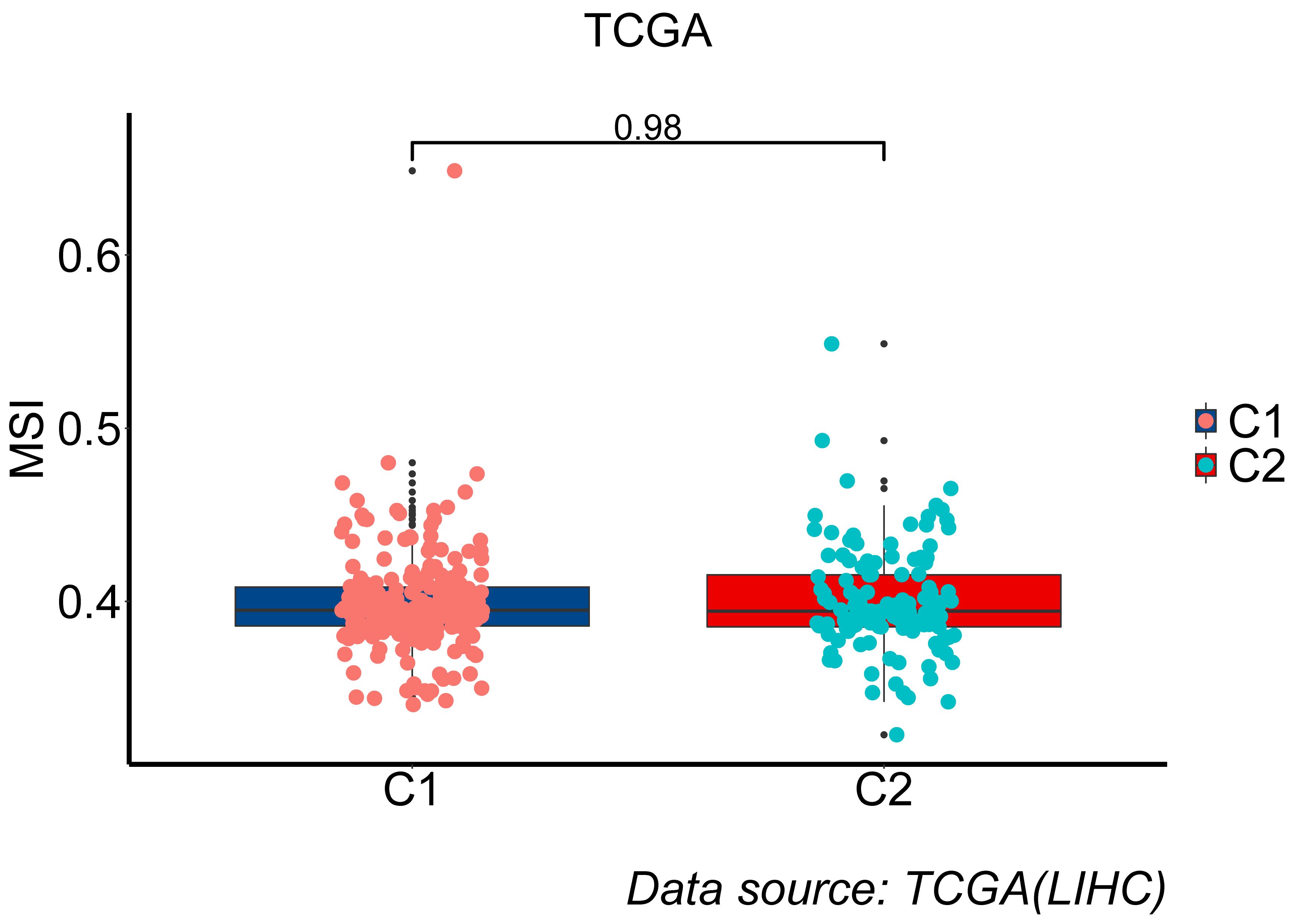

Supplement: Supplementary file 6 — Supplementary Figure S5. [file 41598_2023_28436_MOESM6_ESM.tif]

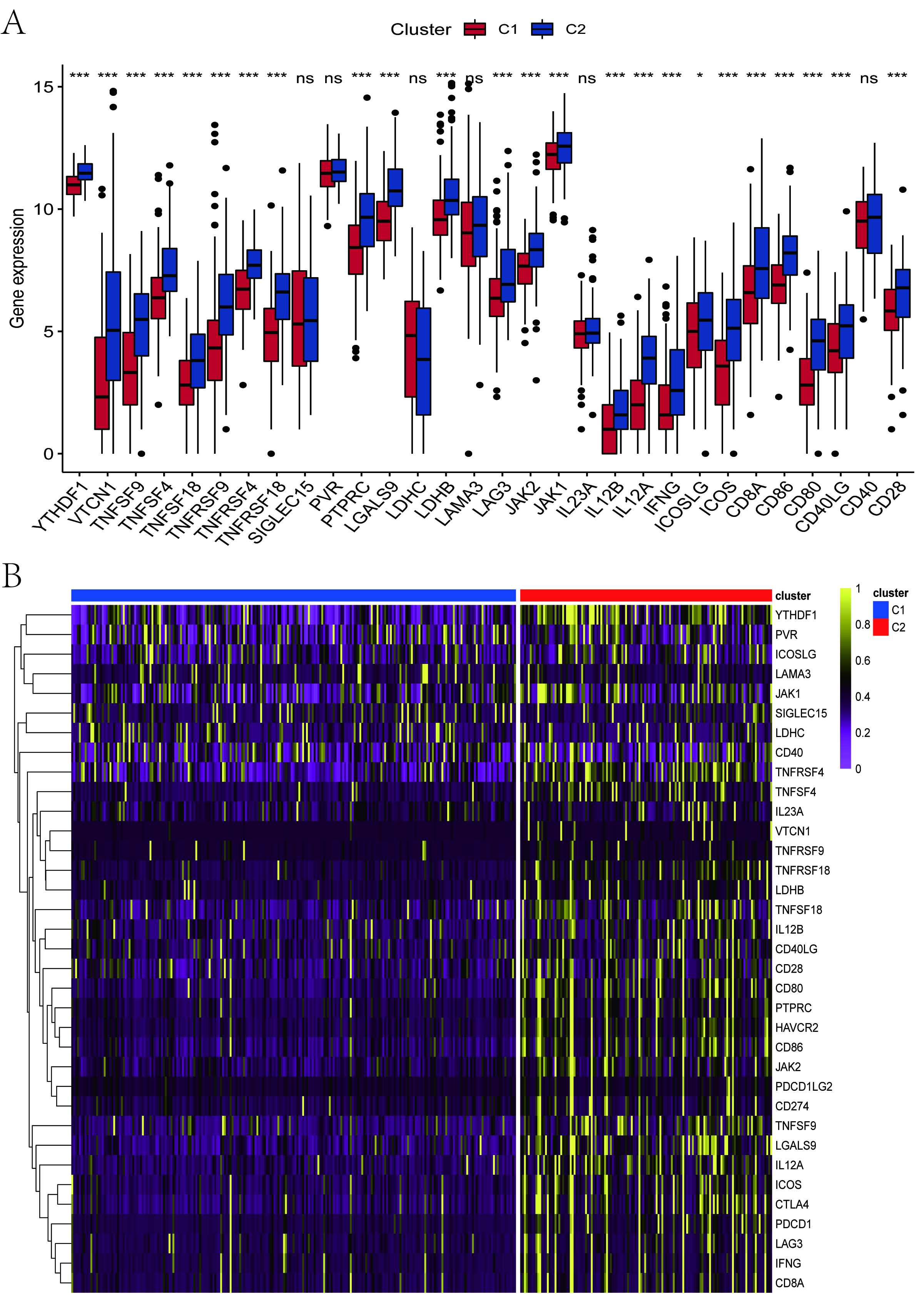

Supplement: Supplementary file 7 — Supplementary Figure S6. [file 41598_2023_28436_MOESM7_ESM.tif]

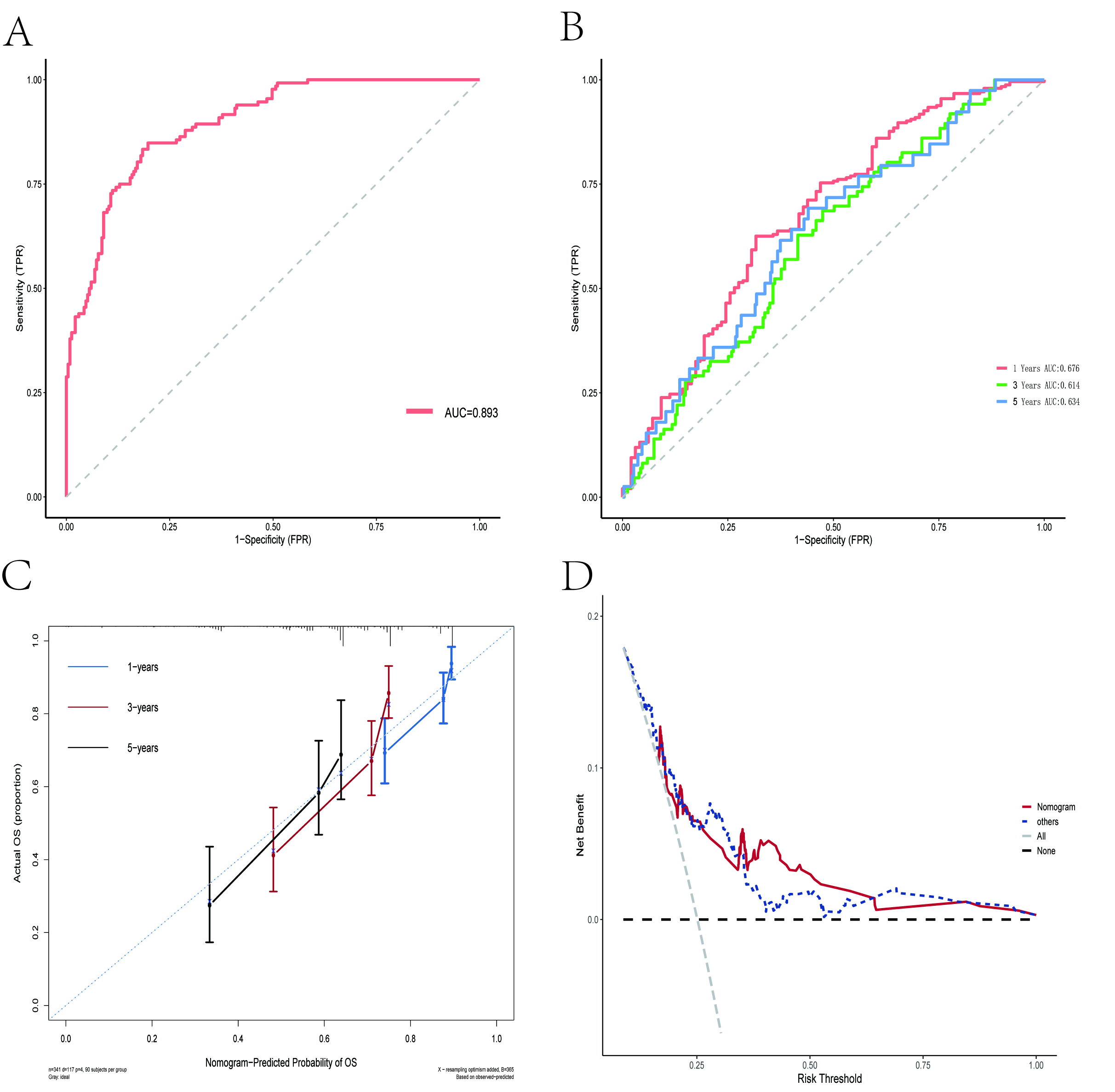

Supplement: Supplementary file 9 — Supplementary Figure S8. [file 41598_2023_28436_MOESM9_ESM.tif]
