# Supplementary material for: Cellular senescence affects energy metabolism, immune infiltration and immunotherapeutic response in hepatocellular carcinoma
Source: Sci Rep. 2023 Jan 20;13:1137. doi: 10.1038/s41598-023-28436-z (PMC9860043; doi:10.1038/s41598-023-28436-z)
Supplement: Supplementary file 10 — Supplementary Tables. [file 41598_2023_28436_MOESM10_ESM.docx]

**Supplement Table 1** Clinical characteristics of HCC patients from 2 independent external validation cohorts

| **Variables** | **GSE14520**  **(n=220)** | **ICGC**  **(n=231)** |
| --- | --- | --- |
| **Age(Year)**  **Median**  **Range** | 50  21-77 | 69  31-89 |
| **Gender** |  |  |
| Male | 190 | 170 |
| Female | 30 | 61 |
| **TNM** |  |  |
| I | 93 | 36 |
| II  III  IV  NA | 77  44  -  2 | 105  71  19  - |
| **BCLC** |  |  |
| 0  A | 20  148 | -  - |
| B  C  NA | 22  28  2 | -  -  - |
| **OS Status** |  |  |
| Alive | 136 | 189 |
| Death | 84 | 42 |
|  |  |  |
